# Ritualized experiences and perceived authenticity in shaping tourists’ willingness to support intangible cultural heritage inheritance

**DOI:** 10.1371/journal.pone.0358987

**Published:** 2026-09-21

**Authors:** Wangbing Liang, Haoyu Song

**Affiliations:** 1 Tourism College of Northwest Normal University, Lanzhou, China; 2 Tourism Development Research Academy of Gansu, Lanzhou, China; Sichuan Agricultural University, CHINA

## Abstract

Intangible cultural heritage tourism plays an important role in cultural sustainability, yet how tourists’ experiential perceptions are associated with their willingness to support ICH inheritance remains insufficiently understood. Taking Gansu Shehuo, a representative form of Chinese folk intangible cultural heritage, as the empirical context, this study investigates how ritualized experiences and perceived authenticity are associated with tourists’willingness to support ICH inheritance.Based on 390 valid questionnaires, the study further explores both the independent effects and configurational pathways underlying tourists’ willingness to support ICH inheritance.The results show that uniqueness, ceremoniality, non-functionality, objective authenticity, and existential authenticity are significantly and positively associated with tourists’ willingness to support ICH inheritance, whereas commitment and constructive authenticity have no significant direct effects. The necessity analysis indicates that no single antecedent condition is necessary for either high or low willingness. The configurational analysis further identifies six configurations leading to high willingness and one configuration leading to low willingness, demonstrating equifinality and causal asymmetry. The findings suggest that sustainable ICH revitalization may benefit from the coordinated development of ritual distinctiveness, symbolic order, historical credibility, and lived authenticity rather than from isolated experiential attributes.

## 1 Introduction

Heritage tourism provides an important context for understanding how cultural resources contribute to preservation, identity formation, and sustainable development [1–4]. Tourists are no longer regarded solely as passive consumers of heritage, because their interpretation, communication, and post-visit behaviour can influence the circulation and continuation of cultural meanings [5]. This role is particularly relevant to intangible cultural heritage, whose continuity depends on living practice, performance, participation, and interaction as well as institutional and community protection.

Following Ruan et al. [6], the construct of tourists’ willingness to support ICH inheritance was adapted to the context of intangible cultural heritage tourism. In the present study, tourists’ willingness to support ICH inheritance refers to tourists’ intention to contribute to the continuity of ICH through cultural communication, continued attention, participation in inheritance-related activities, and the contribution of time or financial resources. It does not imply that tourists replace local communities or heritage bearers, nor does it denote the actual mastery and intergenerational transmission of heritage knowledge or skills. Rather, it reflects the complementary role that tourists may play in supporting ICH continuity.

In heritage tourism, tourists construct cultural meanings through interpretation, interaction, emotional engagement, and connections between heritage and personal identity [7–9]. Consequently, their willingness to support intangible cultural heritage cannot be explained solely by the visible content or entertainment value of heritage activities, but must also be understood through the symbolic and emotional meanings generated during the experience.

Despite the growing recognition of tourists’ supportive role, the experiential mechanisms through which intangible cultural heritage tourism supports cultural continuity remain insufficiently explained. Authenticity is widely recognized as a central but multidimensional and context-dependent concept in heritage tourism [10,11], while less is known about how ritualized experiences communicate cultural distinctiveness, symbolic order, and transmission value in living heritage settings [12–14]. Ritualized experiences describe how cultural meanings are enacted through distinctive procedures, symbolic order, bodily participation, and non-utilitarian practices [15], whereas perceived authenticity reflects how tourists evaluate the historical credibility, socially constructed meaning, and lived genuineness of these encounters [16,17]. Integrating these constructs can therefore connect the performative organization of intangible cultural heritage experiences with tourists’ cognitive and emotional interpretations of heritage meanings.

Although ritualized experiences and perceived authenticity are conceptually distinct, they may also be theoretically related. Ritualized procedures, symbolic order, and culturally distinctive performances may provide experiential cues through which tourists form judgments of authenticity. However, the present study does not assume a fixed causal ordering between these constructs. Instead, it treats them as complementary experiential domains and examines their differentiated net associations and configurational combinations in relation to tourists’ willingness to support ICH inheritance.

However, limited attention has been paid to how ritualized experiences and perceived authenticity jointly shape tourists’ willingness to support ICH inheritance. Existing integrative studies have mainly examined market-oriented outcomes, such as visit intention, satisfaction, and destination image, and have often treated experiential antecedents as broad aggregate constructs rather than as differentiated dimensions [18]. Accordingly, a more experience-based and sustainability-oriented perspective is needed to examine how ritualized experiences and perceived authenticity, both independently and configurationally, shape culturally oriented behavioural outcomes in ICH tourism.

Against this background, Gansu Shehuo provides a suitable empirical context for examining the relationship between ICH tourism experiences and heritage transmission. As a representative form of Chinese folk ICH, Gansu Shehuo combines local distinctiveness, ritualized performance, collective participation, and symbolic expression. It integrates ritual procedures, folk performance, local cultural symbols, and community interaction, thereby enabling tourists to perceive both ritual distinctiveness and authenticity [19]. These characteristics make Gansu Shehuo an appropriate setting for exploring how tourists’ experiential perceptions are translated into their willingness to support intangible cultural heritage, a culturally oriented behavioural outcome closely related to sustainable ICH transmission [6].

Building on the above discussion, this study uses Gansu Shehuo as a representative folklore-based ICH tourism context to examine the associations of ritualized experiences and perceived authenticity with tourists’ willingness to support ICH inheritance. Specifically, this study has four objectives: first, to examine the dimensions of ritualized experiences and perceived authenticity in folk ICH tourism and assess their net associations with tourists’ willingness to support ICH inheritance; second, to determine whether any of these dimensions constitute necessary conditions for this willingness; third, to uncover the configurational pathways associated with high willingness; and fourth, to combine necessity analysis with fsQCA to explain asymmetric configurational relationships between antecedent conditions and culturally oriented behavioral outcomes.By addressing these objectives, this study advances existing research in three respects. First, it integrates ritualized experiences and perceived authenticity as complementary mechanisms linking the performative presentation of living heritage with tourists’ interpretation of its credibility and meaning. Second, it extends the outcome domain of heritage tourism research from conventional market-oriented responses to tourists’ willingness to support ICH inheritance, thereby establishing a more direct connection between tourism experience and cultural sustainability. Third, by combining SEM and fsQCA, it explains both the differentiated net effects of individual experiential dimensions and the alternative configurations through which ritualized and authentic experiences jointly generate heritage-supportive intentions [20,21].

## 2 Literature review and hypotheses development

### 2.1 Ritualized experiences and tourists’ willingness to support ICH inheritance

The essence of intangible cultural heritage (ICH) tourism lies not merely in the display of cultural content, but also in whether tourists can develop symbolic perception, procedural awareness, and emotional engagement that distinguish the experience from everyday life [9]. Compared with general sightseeing, ICH tourism relies more heavily on live performance, contextual staging, and on-site interaction to communicate cultural meanings and support the sustainable transmission of living traditions. In this regard, ritualized experiences provide an important lens through which to understand how tourists perceive, interpret, and evaluate ICH tourism [22]. Existing research suggests that ritualized experiences in ICH tourism are multidimensional, primarily encompassing uniqueness, commitment, ceremoniality, and non-functionality, and that these dimensions jointly shape tourists’ understanding and appreciation of heritage culture [19]. Moreover, ritualized experiences are not generated by the activity itself alone, but are co-constructed by organizers, performers, local communities, and tourists, and are reflected in the atmosphere of the setting, local symbols, bodily participation, and interactive relations [8]. From a broader ICH and cultural sustainability perspective, tourists’ perceptions of cultural distinctiveness, symbolic order, and cultural significance may further influence their support for, engagement with, and willingness to support intangible cultural heritage [23–25].

Ritualized experiences may influence not only whether tourists participate in ICH activities, but also how they interpret cultural meanings and whether they are willing to support cultural continuity. Prior research has shown that interactivity, immersion, and emotional involvement in on-site experiences can enhance tourists’ memorable experiences and behavioural responses, while narrative contexts, self-congruity, cultural labels, and local cultural atmospheres can further shape cultural identity, purchase intention, and other post-experience outcomes [26–28]. Research on destination attachment and place identity likewise suggests that the emotional and cognitive bonds formed in tourism settings often provide an important basis for tourists’ subsequent support, communication, and transmission-related behaviours [29]. In the context of folklore-based ICH tourism, uniqueness enables tourists to recognise that the experience differs from ordinary festive events and routine sightseeing [30]; commitment strengthens psychological and behavioural investment; ceremoniality highlights symbolic order and ritual atmosphere [31]; and non-functionality helps tourists perceive cultural significance beyond entertainment and consumption. It can therefore be expected that stronger ritualized experiences are associated with a higher willingness to support ICH inheritance.

Based on the above discussion, ritualized experiences in folklore-based ICH tourism can be conceptualised as a multidimensional construct comprising uniqueness, commitment, ceremoniality, and non-functionality. As these dimensions shape tourists’ perceptions of cultural distinctiveness, symbolic order, and cultural significance,they are expected to be positively associated with tourists’ willingness to support ICH inheritance.

Accordingly, the following hypotheses are proposed.

**Hypothesis (H1a):** Uniqueness is positively associated with tourists’ willingness to support ICH inheritance.

**Hypothesis (H1b):** Commitment is positively associated with tourists’ willingness to support ICH inheritance.

**Hypothesis (H1c):** Ceremoniality is positively associated with tourists’ willingness to support ICH inheritance.

**Hypothesis (H1d):** Non-functionality is positively associated with tourists’ willingness to support ICH inheritance.

### 2.2 Perceived authenticity and tourists’ willingness to support culture

Perceived authenticity has long been regarded as one of the most central and debated concepts in heritage tourism research [32]. In heritage and ICH tourism, authenticity matters not only because it shapes tourists’ evaluations of heritage resources, but also because it provides an important basis for understanding how visitors interpret cultural meanings and develop subsequent attitudes and behavioral responses [33]. Existing studies generally conceptualize perceived authenticity as a multidimensional construct, rather than a single static attribute. In particular, Wang argues that authenticity in tourism should not be understood solely in relation to objects, but also in relation to socially constructed meanings and tourists’ lived experiences [34]. Building on this perspective, perceived authenticity is commonly discussed in terms of objective authenticity, constructive authenticity, and existential authenticity [35,36]. This perspective is especially relevant to ICH tourism, where tourists encounter not merely static heritage objects, but living cultural practices involving performances, narratives, skills, rituals, heritage bearers, and local atmospheres [37]. In such contexts, perceived authenticity becomes a key lens through which tourists judge whether a cultural experience is credible, meaningful, and worthy of continued attention and support for sustainable transmission [38].

The three dimensions of perceived authenticity may operate through different mechanisms in shaping tourists’ responses to heritage and ICH experiences. Objective authenticity primarily concerns tourists’ perceptions of the historical basis, traditional style, and cultural credibility of heritage expressions, thereby strengthening their trust in the heritage resource itself [39]. Constructive authenticity, by contrast, is more closely associated with tourists’ recognition of staged settings, interpretive narratives, and symbolic representations, through which cultural meanings are socially negotiated and experienced. Existential authenticity places greater emphasis on tourists’ sense of presence, self-realization, and emotional resonance during the experience. Prior studies have shown that these dimensions do not contribute equally to post-visit outcomes [40]. Objective authenticity often reinforces tourists’ evaluations of cultural value and destination credibility, whereas existential authenticity is more directly associated with emotional attachment, loyalty, and behavioral tendencies [41–43]. Meanwhile, authenticity may also influence tourist outcomes indirectly through experience quality, involvement, nostalgia, and satisfaction [44–47]. Taken together, these findings suggest that, in ICH tourism, different dimensions of perceived authenticity are likely to show differentiated associations with tourists’ willingness to support ICH inheritance.

Recent studies have shown that authenticity is related not only to traditional outcomes such as satisfaction, loyalty, and visit intention, but also to higher-order heritage-oriented outcomes, including heritage responsibility, place attachment, and long-term support for heritage sites [48–50]. This line of research is further supported by evidence showing that destination authenticity can reinforce place attachment and loyalty in heritage-related tourism settings [51]. This suggests that, in ICH tourism, perceived authenticity may function as an important mechanism through which tourists develop deeper emotional, moral, and behavioral connections with heritage [52]. When tourists are able to recognize the historical credibility and cultural value of ICH expressions while also experiencing a strong sense of presence and resonance, they may be more likely to regard such culture as worthy of understanding, supporting, and passing on. Accordingly, perceived authenticity is likely to play an important role in shaping tourists’ willingness to support intangible cultural heritage, thereby contributing to sustainable ICH transmission.

Based on the above discussion, perceived authenticity in ICH tourism can be understood as a multidimensional construct comprising objective authenticity, constructive authenticity, and existential authenticity. As these dimensions reflect tourists’ evaluations of cultural credibility, symbolic meaning, and lived experience, they are expected to positively influence tourists’ willingness to support intangible cultural heritage. Accordingly, the following hypotheses are proposed.

**Hypothesis (H2a):** Objective authenticity is positively associated with tourists’ willingness to support ICH inheritance.

**Hypothesis (H2b):** Constructive authenticity is positively associated with tourists’willingness to support ICH inheritance.

**Hypothesis (H2c):** Existential authenticity is positively associated with tourists’willingness to support ICH inheritance.

### 2.3 Complexity theory, ritualized experiences, and perceived authenticity

Higher-order outcomes in heritage and ICH tourism are rarely associated with a single antecedent condition alone. Instead, they are more likely to be associated with combinations of experiential, cognitive, and emotional factors.Existing research suggests that outcomes such as experience quality, satisfaction, destination loyalty, place attachment, and supportive behaviour are often shaped by combinations of antecedent conditions rather than by isolated effects [19,53–55]. From the perspective of complexity theory, antecedent conditions may be complementary, substitutable, and context-dependent, and high and low outcomes should not be assumed to mirror one another [20]. Therefore, relying solely on symmetric approaches may overlook important combinations of conditions that contribute to tourists’ willingness to support intangible cultural heritage.

This configurational perspective is particularly relevant to ICH tourism, where ritualized experiences and perceived authenticity are inherently intertwined. Ritualized experiences highlight cultural distinctiveness, symbolic order, and non-utilitarian meaning, whereas perceived authenticity shapes tourists’ judgements of cultural credibility, emotional resonance, and experiential value. In such contexts, high willingness to support intangible cultural heritage may not depend on all dimensions being simultaneously strong, but may instead result from different combinations of key ritualized and authenticity-related conditions. Recent heritage tourism research has increasingly used configurational logic to explain how multiple antecedents jointly shape heritage-related responsibility, support, and behavioural outcomes [21,24,49]. Accordingly, examining the joint effects of ritualized experiences and perceived authenticity can offer a more nuanced understanding of how tourists develop willingness to support culture in folklore-based ICH tourism.

Based on the above discussion, this study argues that tourists’ willingness to support culture is not merely the result of the independent effects of ritualized experiences or perceived authenticity, but may also be shaped by their combined configurations. Accordingly, the following hypothesis is proposed.

**Hypothesis (H3):** Different configurations of ritualized experiences and perceived authenticity constitute sufficient pathways associated with both high and low tourists’ willingness to support ICH inheritance.

**Fig 1:**
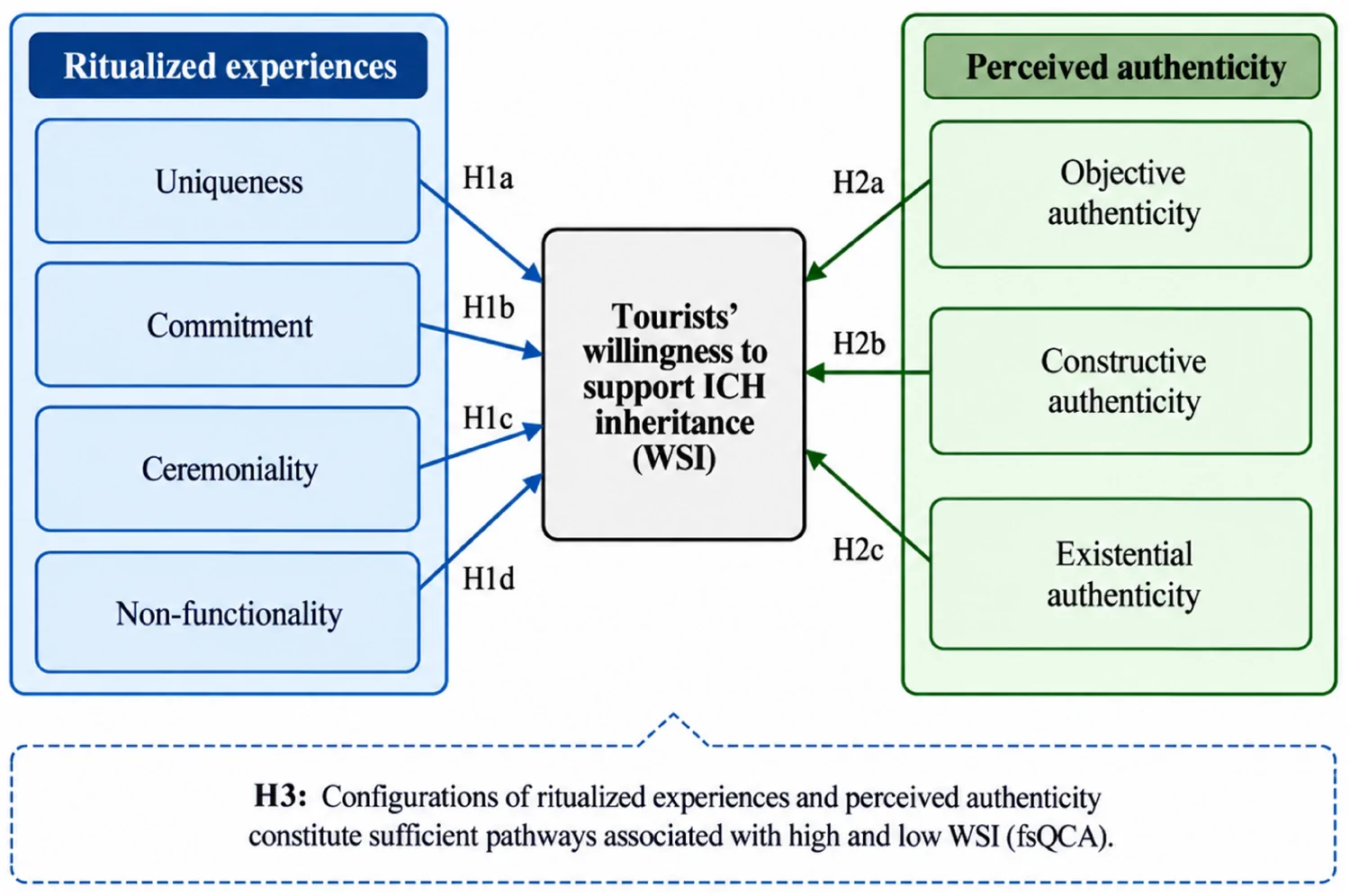
Conceptual model.

The proposed research framework is shown in Fig 1.

## 3 Research design and data collection

### 3.1 Case context

Gansu Shehuo was selected as the empirical context of this study for three main reasons. First, as a representative form of living folk intangible cultural heritage in China, Shehuo has been relatively well preserved in Gansu and embodies strong historical continuity, local cultural distinctiveness, and collective participation. Second, Gansu Shehuo integrates ritual procedures, folk performance, local symbols, and community interaction, thereby creating favourable conditions for tourists to perceive ritualized experiences and perceived authenticity. Third, Shehuo activities in Yongdeng County, Tongwei County, and Tianshui are well established and locally distinctive. Together, these sites not only reflect the common characteristics of Gansu Shehuo, but also reveal experiential variations across different settings. As such, they provide an appropriate context for examining how tourists develop willingness to support intangible cultural heritage in a folklore-based ICH tourism setting.

### 3.2 Measurement scales

All constructs in this study were adapted from established scales and adjusted to the context of Gansu Shehuo while retaining their original conceptual meanings. The original English items were translated into Chinese and then independently back-translated into English. Differences between the original and back-translated versions were discussed and revised to ensure semantic equivalence. Subsequently, experts in tourism management from Gansu Province evaluated the clarity, conceptual consistency, and contextual relevance of the items. A pilot survey was then conducted among tourists to identify ambiguous wording and comprehension difficulties. Based on the expert review and pilot feedback, minor wording adjustments were made before the formal survey.

Ritualized experiences were measured across four dimensions: uniqueness, commitment, ceremoniality, and non-functionality, mainly adapted from Yan et al. [19]. Perceived authenticity was measured through three dimensions: objective authenticity, constructive authenticity, and existential authenticity, drawing on established authenticity research in heritage tourism and revised with reference to Chinese heritage tourism contexts [36,47]. Tourists’ willingness to support ICH inheritance was measured using four items adapted from Ruan et al. [6], covering recommendation, continued attention, participation, and willingness to contribute time or financial resources. Although these items reflect different forms of supportive intention, they represent a common underlying orientation toward supporting the continuity of ICH rather than actual heritage inheritance or intergenerational transmission behavior. Specifically, recommendation and continued attention reflect communicative and attentional support, participation reflects engagement intention, and the contribution of time or financial resources reflects resource-based support. Together, these indicators capture complementary manifestations of a broader willingness to support ICH inheritance. The final questionnaire consisted of four items for uniqueness, three for commitment, four for ceremoniality, three for non-functionality, three for objective authenticity, three for constructive authenticity, three for existential authenticity, and four for tourists’ willingness to support intangible cultural heritage. Except for demographic variables, all items were measured on a seven-point Likert scale ranging from 1 = “strongly disagree” to 7 = “strongly agree”.

To reduce potential common method bias, respondents were assured of anonymity and confidentiality, and the questionnaire items were expressed in concise and context-specific language to reduce ambiguity.

### 3.3 Data collection

Data were collected on site from November 18, 2025 to January 8, 2026 at the venues where Shehuo performances were staged in Yongdeng County, Tongwei County, and Tianshui. An on-site non-probability sampling strategy was used to recruit tourists who had just watched a Shehuo performance or participated in related experiential activities. Before the questionnaires were administered, the research team briefly introduced the purpose of the study and assured respondents that their personal information would remain confidential and that the data would be used for academic research only. Written informed consent was obtained from all participants before questionnaire completion. To improve response quality, each questionnaire was completed under the supervision of the research team, who were available to clarify any questions raised by respondents. A total of 425 questionnaires were collected, including 125 in Yongdeng County, 198 in Tongwei County, and 102 in Tianshui. Of these, 390 were retained as valid, yielding an effective response rate of 91.8%. To assess sample adequacy, the final sample of 390 exceeded the commonly used ratio of 10 observations per measured item for SEM, which would require a minimum of 270 cases for the 27 observed items in this study.The demographic profile of the valid respondents, including gender, age, education, income, occupation, and prior experience with Shehuo, is summarized in Table 1.

**Table 1:** Sample demographics characteristic.

Basic feature	Sample group	Frequency	Percentage (%)	Basic feature	Sample group	Frequency	Percentage (%)
Gender	Female	182	46.7	Average monthly income	Under 3,000RMB	85	21.8
	Male	208	53.3		3001-5000RMB	151	38.7
Age	18-25 years old	150	38.5		5001-8000RMB	115	29.5
	26-30 years old	121	31.0		Over 8001 RMB	39	10.0
	31-40 years old	70	18.0	Occupation type	Institutional organization/Civil servant	21	5.4
	41-50 years old	27	6.9		Company staff	52	13.3
	51-60 years old	22	5.6		Full-time self-employed	57	14.6
Education level	Less than community-college degree	73	18.7		Student	162	41.5
	Bachelor’s degree	249	63.9		Retiree	28	7.2
	Master’s degree	62	15.9		Other	70	18.0
	Doctorate or above	6	1.5	Prior visit(s) frequency	1 time	84	21.6
					2 times	176	45.1
					3 times or above	130	33.3

Because all variables were measured using self-reported questionnaires, common method bias was assessed before evaluating the measurement model. Harman’s single-factor test extracted seven factors with eigenvalues greater than 1, and the first unrotated factor explained 35.21% of the total variance, below the 50% threshold. In addition, the single-factor measurement model showed a substantially poorer fit (χ²/df = 9.391, CFI = 0.544, TLI = 0.506, and RMSEA = 0.147) than the proposed eight-factor model (χ²/df = 1.100, CFI = 0.995, TLI = 0.994, and RMSEA = 0.016). Taken together, these results suggest that common method bias is unlikely to dominate the observed relationships; however, they do not completely rule out the possibility of shared-method variance.

Following the conventional two-stage SEM procedure, the measurement model was first assessed in terms of internal consistency, convergent validity, and discriminant validity. As shown in Table 2, all retained indicators exhibited acceptable standardised loadings above 0.70. Cronbach’s α values ranged from 0.794 to 0.888, composite reliability (CR) values ranged from 0.845 to 0.889, and the average variance extracted (AVE) values ranged from 0.645 to 0.672, all exceeding the recommended thresholds. These results indicate satisfactory internal consistency and convergent validity of the constructs. As shown in Table 3, the square roots of the AVEs for all constructs were greater than the corresponding inter-construct correlations, thereby supporting discriminant validity. Overall, the measurement model demonstrated satisfactory reliability and validity and provided a sound basis for subsequent structural model testing.

**Table 2:** Reliability test of the scale.

Construct/items	Normalized load factor	Cronbach’s α	AVE	CR
**Uniqueness**		0.888	0.667	0.889
UNQ1 Some of the props, costumes, and movements in the Shehuo performances carry unique cultural meanings.	0.812			
UNQ2 This experience holds a distinctive meaning for me, rather than being merely a tourism activity.	0.849			
UNQ3 The surrounding environment creates a unique atmosphere for me, such as solemnity or festivity.	0.848			
UNQ4 Shehuo makes me perceive the distinctiveness of the ritual.	0.753			
**Commitment**		0.850	0.655	0.850
COM1 I approach each ritual element of the Shehuo event with great care and attentiveness.	0.800			
COM2 I am willing to devote time and energy to participating in or watching Shehuo.	0.842			
COM3 Throughout the Shehuo event, I remain serious and attentive.	0.784			
**Ceremoniality**		0.884	0.656	0.884
CER1 I feel that I am participating in Shehuo within a formal ritual setting.	0.786			
CER2 The entire Shehuo process embodies the spirit of cultural inheritance.	0.829			
CER3 The props and visual elements used in Shehuo convey a strong sense of ritual.	0.814			
CER4 I can perceive the distinctive local etiquette embedded in the Shehuo event.	0.811			
**Nonfunctionality**		0.852	0.657	0.852
NF1 Some procedures in the Shehuo event are not intended for practical purposes, but rather to fulfil ritual requirements.	0.801			
NF2 Even when certain parts of the Shehuo event are not strictly necessary, they are still preserved in full.	0.828			
NF3 Some procedures in the Shehuo event are rather elaborate and time-consuming.	0.803			
**Objective authenticity**		0.844	0.645	0.845
OA1 The performance form of Shehuo retains its original authentic character.	0.782			
OA2 Shehuo performances display a strong local traditional style.	0.813			
OA3 Shehuo performances have a long history, and their content is grounded in historical facts.	0.814			
**Constructive authenticity**		0.859	0.672	0.860
CA1 The atmosphere at the Shehuo event makes me feel as if I were immersed in a traditional festival of the past.	0.818			
CA2 The Shehuo performances, drumbeats and gongs, formation displays, and on-site interactions are highly evocative.	0.861			
CA3 The setting and performance atmosphere of the Shehuo event make me genuinely feel the historical continuity of local folk culture.	0.779			
**Existential authenticity**		0.847	0.650	0.848
EA1 I can feel the warmth of local people and naturally become part of the experience.	0.820			
EA2 The Shehuo performances give me a deeper understanding of intangible cultural heritage.	0.819			
EA3 I thoroughly enjoy this unique traditional and spiritual experience.	0.779			
**Tourists’ willingness to support ICH inheritance**		0.886	0.660	0.886
WSI1 I am willing to recommend traditional culture to others.	0.794			
WSI2 I am willing to actively focus on the protection and inheritance of Shehuo culture.	0.824			
WSI3 I am willing to participate in activities related to the inheritance of Shehuo culture.	0.807			
WSI4 I am willing to devote time and money to preserving and inheriting Shehuo culture.	0.824			

**Table 3:** Discriminative validity and the correlations of variables.

Variables	Uniqueness	Commitment	Ceremoniality	Nonfunctionality	Objective authenticity	Constructive authenticity	Existential authenticity	Tourists’ willingness to support ICH inheritance
Uniqueness	**0.817**							
Commitment	0.425	**0.809**						
Ceremoniality	0.396	0.403	**0.810**					
Nonfunctionality	0.353	0.362	0.383	**0.811**				
Objective authenticity	0.312	0.298	0.318	0.334	**0.803**			
Constructive authenticity	0.209	0.216	0.316	0.275	0.474	**0.820**		
Existential authenticity	0.216	0.322	0.301	0.312	0.388	0.346	**0.806**	
Tourists’ willingness to support ICH inheritance	0.531	0.453	0.545	0.497	0.496	0.403	0.494	**0.812**

### 3.4 Data analysis strategy

This study adopted a combined symmetric–asymmetric analytical strategy to examine the associations of ritualized experiences and perceived authenticity with tourists’ willingness to support ICH inheritance. First, structural equation modelling (SEM) was employed to estimate the net associations of each dimension of ritualized experiences and perceived authenticity with tourists’ willingness to support ICH inheritance. The SEM analysis was conducted in two stages. The measurement model was first evaluated in terms of internal consistency, convergent validity, and discriminant validity. The structural model was then assessed to examine the significance of the hypothesised relationships among the study variables. The SEM analysis was performed using SPSS 27 and AMOS 29, and model fit was evaluated based on conventional indices, including χ²/df, CFI, TLI, and RMSEA.

For the fsQCA procedure, this study adopted the direct calibration method. Based on the original distribution of each variable, the 95%, 50%, and 5% percentiles were set as the thresholds for full membership, the crossover point, and full non-membership, respectively. The condition and outcome variables were then transformed into fuzzy-set membership scores ranging from 0 to 1 [56]. Before conducting the configurational analysis, a necessity analysis was performed for each condition and its negated set, with the consistency threshold set at 0.90. When constructing the truth table, the case frequency threshold was set at 1, the raw consistency threshold at 0.80, and the PRI consistency threshold at 0.75. These criteria were used to exclude contradictory configurations that simultaneously supported both the presence and absence of the outcome [57].

To assess the robustness of the configurational findings, sensitivity analyses were conducted using alternative calibration anchors and more stringent truth-table thresholds. Specifically, the calibration anchors were changed to the 90th, 50th, and 10th percentiles. The case-frequency threshold was subsequently increased from 1 to 3 and further to 5, while the raw-consistency threshold was increased from 0.80 to 0.85; the PRI-consistency threshold was retained at 0.75. The resulting solutions were compared in terms of their substantive configurational patterns and the stability of the high- and low-outcome solutions.

## 4 Results

### 4.1 Structural model results

**Fig 2:**
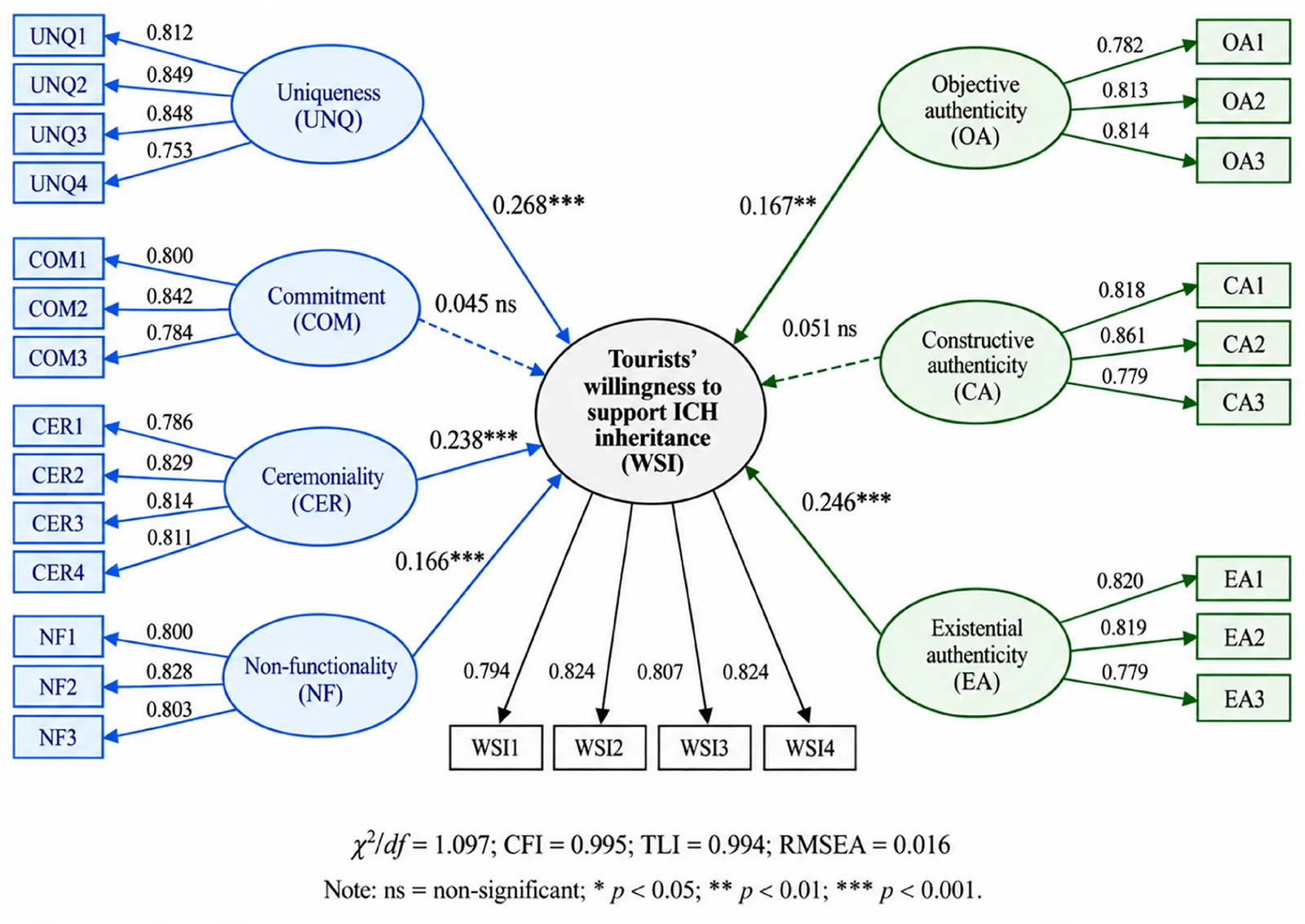
The results of symmetrical modeling using SEM for identifying the sufficient antecedents.

The structural model was subsequently assessed to examine the net associations of ritualized experiences and perceived authenticity with tourists’ willingness to support ICH inheritance. The model showed a satisfactory fit to the data, with χ²/df = 1.097, CFI = 0.995, TLI = 0.994, and RMSEA = 0.016. The structural model results are presented in Fig 2. As shown in Table 4, uniqueness (β = 0.268, p < 0.001), ceremoniality (β = 0.238, p < 0.001), non-functionality (β = 0.166, p < 0.001), objective authenticity (β = 0.167, p = 0.002), and existential authenticity (β = 0.246, p < 0.001) were positively and significantly associated with tourists’ willingness to support ICH inheritance, supporting H1a, H1c, H1d, H2a, and H2c. In contrast, commitment (β = 0.045, p = 0.376) and constructive authenticity (β = 0.051, p = 0.309) were not significantly associated with the outcome, and H1b and H2b were therefore not supported. Overall, the results indicate heterogeneous net associations across the dimensions of ritualized experiences and perceived authenticity.

**Table 4:** Structural model results and hypothesis testing.

	β	S.E.	t-value	p-value	Results
**UNQ → WSI**	0.268	0.059	5.390	<0.001	Supported
**COM → WSI**	0.045	0.053	0.885	0.376	Not supported
**CER → WSI**	0.238	0.050	4.744	<0.001	Supported
**NF → WSI**	0.166	0.052	3.393	<0.001	Supported
**OA → WSI**	0.167	0.054	3.044	0.002	Supported
**CA → WSI**	0.051	0.053	1.017	0.309	Not supported
**EA → WSI**	0.246	0.055	4.948	<0.001	Supported

Note: UNQ = uniqueness; COM = commitment; CER = ceremoniality; NF = non-functionality; OA = objective authenticity; CA = constructive authenticity; EA = existential authenticity; WSI = tourists’ willingness to support intangible cultural heritage.

### 4.2 Necessary condition analysis

Before conducting the configurational analysis, a necessity analysis was performed for each antecedent condition. Following the standard fsQCA criterion, a condition is generally considered necessary when its consistency exceeds 0.90. As shown in Table 5, none of the antecedent conditions, nor their negations, reached this threshold for either high or low tourists’ willingness to support intangible cultural heritage. For high willingness to support intangible cultural heritage, the consistency values of the presence of the antecedent conditions ranged from 0.700 to 0.777. Among them, objective authenticity (0.777), commitment (0.748), and constructive authenticity (0.742) showed relatively high consistency, but none qualified as a necessary condition. For low willingness to support intangible cultural heritage, the consistency values of the absence of the antecedent conditions were comparatively higher overall, with the absence of uniqueness (0.807) and the absence of existential authenticity (0.805) showing the highest values; however, these still fell below the 0.90 threshold. Overall, the results indicate that no single antecedent condition constitutes a necessary condition for either high or low tourists’ willingness to support ICH inheritance. Instead, the outcome appears to be associated with combinations of multiple dimensions of ritualized experiences and perceived authenticity.

**Table 5:** Necessary conditions analysis.

Antecedent conditions	High willingness to support ICH inheritance		Antecedent conditions	Low willingness to support ICH inheritance	
	Consistency	Coverage		Consistency	Coverage
UNQ	0.731	0.814	UNQ	0.524	0.505
~UNQ	0.555	0.574	~UNQ	0.807	0.723
COM	0.748	0.769	COM	0.598	0.533
~COM	0.546	0.610	~COM	0.741	0.719
CER	0.741	0.797	CER	0.545	0.508
~CER	0.542	0.579	~CER	0.783	0.724
NF	0.736	0.791	NF	0.567	0.528
~NF	0.561	0.599	~NF	0.776	0.718
OA	0.777	0.759	OA	0.623	0.527
~OA	0.516	0.612	~OA	0.716	0.736
CA	0.742	0.751	CA	0.614	0.539
~CA	0.545	0.620	~CA	0.717	0.706
EA	0.700	0.806	EA	0.529	0.528
~EA	0.590	0.591	~EA	0.805	0.700

Note: ~ indicates the absence of a condition; UNQ = uniqueness; COM = commitment; CER = ceremoniality; NF = non-functionality; OA = objective authenticity; CA = constructive authenticity; EA = existential authenticity; WSI = tourists’ willingness to support intangible cultural heritage.

### 4.3 Sufficient configurations

The configurational analysis identified six pathways associated with high tourists’ willingness to support intangible cultural heritage (H1–H6) and one pathway associated with low willingness (L1). As shown in Table 6, the overall consistency and coverage of the high-outcome solution were 0.945 and 0.577, respectively, whereas those of the low-outcome solution were 0.951 and 0.358. These results indicate that the identified configurations have satisfactory explanatory power and provide meaningful configurational solutions associated with both high and low tourists’willingness to support ICH inheritance.

**Table 6:** Configurational paths of cultural willingness.

Configuration	High willingness to support ICH inheritance						Low willingness to support ICH inheritance
	H1	H2	H3	H4	H5	H6	L1
UNQ	●	●	●	●	●		⊗
COM	●	●	○	●		●	⊗
CER	●	●	●	●		●	⊗
NF	●	●	●		●	●	⊙
OA	●			●	○	●	⊗
CA		●			●	○	⊗
EA			●	●	●	●	⊗
Raw coverage	0.419	0.404	0.393	0.399	0.398	0.367	0.358
Unique coverage	0.009	0.009	0.007	0.031	0.062	0.031	0.358
Consistency	0.968	0.966	0.970	0.974	0.971	0.966	0.951
Overall coverage	0.577						0.358
Overall consistency	0.945						0.951

Note: ~ indicates the absence of a condition; ● indicates the presence of a core condition; ⊗ indicates the absence of a core condition; ○ indicates the presence of a peripheral condition; ⊙ indicates the absence of a peripheral condition; UNQ = uniqueness; COM = commitment; CER = ceremoniality; NF = non-functionality; OA = objective authenticity; CA = constructive authenticity; EA = existential authenticity; WSI = tourists’ willingness to support ICH inheritance.

Among the configurations associated with high willingness to support ICH inheritance, H1 and H2 may be regarded as ritual-dominant pathways. Both include uniqueness, commitment, ceremoniality, and non-functionality as core conditions, while differing in the salient authenticity condition: objective authenticity in H1 and constructive authenticity in H2. H3 similarly reflects a ritual-oriented pathway in which uniqueness, ceremoniality, non-functionality, and existential authenticity form a configuration associated with high willingness, with commitment as a peripheral condition.

H4–H6 represent more integrated pathways combining ritualized experiences and perceived authenticity. H4 includes uniqueness, commitment, ceremoniality, objective authenticity, and existential authenticity; H5 combines uniqueness, non-functionality, constructive authenticity, and existential authenticity; and H6 combines commitment, ceremoniality, non-functionality, objective authenticity, and existential authenticity. H5 has the highest unique coverage (0.062), indicating that it covers the largest proportion of cases not covered by the other high-outcome configurations. Overall, high willingness to support ICH inheritance is associated with multiple alternative configurations rather than a single uniform pathway.

In contrast, only one configuration (L1) was identified as being associated with low willingness to support ICH inheritance. This configuration is characterised by the concurrent absence of several key experiential conditions, suggesting that low willingness may be associated with a broader deficiency in ritualized-experience and authenticity-related conditions rather than with the absence of any single condition alone. Given that only one low-willingness configuration was identified, this result should be interpreted cautiously; however, its stability was supported by the subsequent robustness assessment.

### 4.4 Robustness assessment

To evaluate the robustness of the fsQCA results, a series of sensitivity analyses were conducted by varying both the calibration anchors and the truth-table thresholds. Specifically, the original calibration anchors based on the 95th, 50th, and 5th percentiles were replaced with the 90th, 50th, and 10th percentiles. The case-frequency threshold was increased from 1 to 3 and, under a more stringent specification, to 5. In addition, the raw-consistency threshold was raised from 0.80 to 0.85, while the PRI-consistency threshold was retained at 0.75.

The alternative specifications yielded substantively consistent configurational patterns. Increasing the case-frequency threshold mainly excluded low-frequency truth-table rows, whereas the more empirically supported configurations associated with both high and low tourists’ willingness to support ICH inheritance remained broadly stable. The low-willingness configuration was particularly stable across specifications, and the overall configurational asymmetry between high- and low-willingness solutions was preserved.

### 4.5 Evaluation through the lens of complexity theory

The results provide support for the complexity perspective adopted in this study. First, the necessity analysis showed that none of the antecedent conditions, nor their negations, reached the consistency threshold of 0.90, indicating that no single condition constitutes a necessary condition for either high or low willingness to support ICH inheritance. Second, the configurational analysis identified multiple distinct pathways associated with high willingness and one pathway associated with low willingness, indicating that different combinations of experiential conditions may be associated with the same outcome.

Third, the configurations associated with high and low willingness are not mirror images of one another. High willingness is associated with several alternative combinations of ritualized experiences and perceived authenticity, whereas low willingness is associated primarily with the concurrent absence of key experiential conditions. This pattern reflects causal asymmetry in the set-theoretic sense. Here, causal asymmetry indicates that configurations associated with the presence of an outcome need not be the inverse of those associated with its absence; it does not imply experimental or temporal causality. Fourth, the role of individual conditions varies across configurations, indicating configurational context dependence rather than a fixed independent association. Overall, these findings support a configurational interpretation of tourists’ willingness to support ICH inheritance.

## 5 Discussion

### 5.1 Discussion of findings

Using Gansu Shehuo as a folklore-based ICH tourism context, this study examined the associations of ritualized experiences and perceived authenticity with tourists’ willingness to support ICH inheritance through the combined use of SEM and fsQCA. The SEM results show that uniqueness, ceremoniality, non-functionality, objective authenticity, and existential authenticity were significantly and positively associated with tourists’willingness to support ICH inheritance, whereas commitment and constructive authenticity showed no significant independent net associations.This finding suggests that tourists’willingness to support ICH inheritance is more strongly associated with the perceived distinctiveness, ritual atmosphere, symbolic meaning, historical credibility, and lived resonance of ICH experiences than with general psychological investment or socially constructed authenticity alone.

The apparent difference between the SEM and fsQCA results should not be interpreted as a contradiction, because the two approaches address different analytical questions. SEM estimates the independent net association of each predictor with the outcome while accounting for the other predictors, whereas fsQCA examines whether conditions become relevant as part of sufficient combinations. Accordingly, the nonsignificant SEM coefficients for commitment and constructive authenticity do not imply that these conditions are irrelevant. Their presence in particular configurations indicates configurational relevance, suggesting that they may contribute to tourists’ willingness to support ICH inheritance when combined with complementary ritualized-experience or authenticity conditions, even though they do not exhibit significant independent net associations.

The necessity analysis further shows that no single antecedent condition constitutes a necessary condition for either high or low willingness. This result indicates that tourists’ willingness to support intangible cultural heritage cannot be explained by any isolated experiential factor. Instead, it reflects a complex causal structure shaped by the interaction of multiple ritualized and authenticity-related conditions.

The configurational analysis identifies six pathways associated with high willingness and one pathway associated with low willingness. This finding demonstrates that high willingness to support intangible cultural heritage can be generated through multiple alternative combinations of ritualized experiences and perceived authenticity, whereas low willingness is mainly associated with the concurrent absence of key experiential conditions. Taken together, these findings suggest that the sustainable revitalization of folklore-based ICH tourism depends not only on strengthening individual experiential attributes, but also on the coordinated construction of ritualized and authentic experiences.

### 5.2 Theoretical implications

This study makes three theoretical contributions to ICH tourism and cultural sustainability.First, it integrates ritualized experiences and perceived authenticity into a unified explanatory framework. Although both constructs have been widely examined in heritage tourism, they represent different but complementary aspects of tourists’ experiences. Ritualized experiences explain how ICH meanings are presented through distinctive procedures, symbolic order, bodily participation, and ceremonial practices, whereas perceived authenticity reflects how tourists evaluate the credibility, cultural meaning, and personal genuineness of these experiences. Their integration therefore connects the performative presentation of ICH with tourists’ interpretation and internalization of heritage meanings.

Second, this study extends previous research by focusing on tourists’ willingness to support ICH inheritance rather than conventional market-oriented outcomes such as satisfaction, loyalty, and revisit intention. Tourists’ willingness to support ICH inheritance reflects their intention to communicate ICH-related cultural meanings, maintain continued attention, participate in inheritance-related activities, and provide time or resource support for the continuity of ICH. This establishes a more direct link between tourism experience and cultural sustainability and highlights tourists’ potential role not only as consumers of heritage experiences but also as supporters of the communication and continuity of living culture. Importantly, this role is complementary rather than substitutive: tourists may contribute through communication, continued attention, participation, and resource support, while local communities and heritage bearers remain the primary agents responsible for cultural practice and intergenerational transmission.This framework is particularly relevant to ICH, which is continuously reproduced through performance, ritual practice, community participation, and situated interaction.

Third, this study provides a complexity-oriented explanation of tourists’ willingness to support ICH inheritance. The SEM results reveal that different dimensions of ritualized experiences and perceived authenticity have heterogeneous net associations, while the fsQCA results show that conditions without significant independent net associations may still be configurationally relevant when combined with other conditions. The identification of multiple pathways associated with high willingness to support ICH inheritance further demonstrates equifinality and configurational asymmetry. By combining SEM and fsQCA, this study moves beyond isolated linear relationships and offers a more nuanced explanation of how ritualized experiences and perceived authenticity jointly relate to tourists’ willingness to support ICH inheritance and, more broadly, to cultural sustainability.

### 5.3 Practical implications

The findings offer practical implications for ICH tourism destinations, event organizers, and heritage managers. First, greater attention should be given to preserving and presenting ritualized experiences.Given that uniqueness, ceremoniality, and non-functionality were significantly associated with tourists’ willingness to support ICH inheritance, managers may consider highlighting the symbolic procedures, traditional costumes, performance rhythms, spatial arrangements, and local cultural markers that distinguish Shehuo from ordinary festive events or sightseeing activities. Ritual elements that may appear non-utilitarian should not be over-simplified or excessively commercialized, as they often carry important symbolic meanings for cultural continuity.

Second, greater attention may be given to both cognitive and experiential authenticity. The significant associations of objective authenticity and existential authenticity with the outcome suggest that tourists’ willingness to support ICH inheritance is related not only to their recognition of historical credibility and cultural value, but also to their sense of presence, resonance, and meaning during participation. Therefore, organizers may consider improving the interpretation of heritage history, local traditions, and performance logic through authentic narratives, representative props, and the involvement of heritage bearers.

Third, the configurational results suggest that different combinations of ritualized experiences and perceived authenticity are associated with high willingness to support ICH inheritance.Therefore, ICH tourism destinations may benefit from avoiding a uniform development model and considering differentiated strategies according to local resources and event settings. At the same time, managers may also need to recognize that low willingness is mainly associated with the concurrent absence of key experiential conditions. Weakening ritual distinctiveness, diluting authenticity, or reducing visitors’ sense of participation may undermine the heritage value and sustainability potential of ICH tourism. Overall, the findings suggest that sustainable ICH revitalization may benefit not only from increasing visibility, but also from fostering deeper cultural understanding and heritage-supportive engagement through high-quality ritualized and authentic experiences.

### 5.4 Limitations and future research

This study has several limitations. First, it focuses on Gansu Shehuo as a single folklore-based ICH tourism context, which may limit the generalisability of the findings to other destinations or ICH types. Second, the on-site non-probability sampling and single-source self-reported data may limit representativeness and introduce response bias, while potential heterogeneity across the three study locations was not explicitly examined. Third, the use of cross-sectional survey data makes it difficult to capture how tourists’ experiences and willingness to support ICH inheritance may change over time. Accordingly, the observed relationships should be interpreted as associations rather than evidence of temporal or causal effects. Future longitudinal or experimental research is needed to clarify the direction and causal ordering of these relationships.

Fourth, this study examines tourists’ willingness to support ICH inheritance as a supportive behavioural intention rather than as actual heritage inheritance or intergenerational transmission behaviour. Future research should examine whether tourists’ communicative, participatory, and resource-based intentions translate into observable support behaviours and how such support interacts with the long-term transmission roles of local communities and heritage bearers. Finally, the model mainly considers ritualized experiences and perceived authenticity, without incorporating other potentially relevant variables such as cultural identity, emotional resonance, or place attachment. Future research could extend the model by incorporating these factors and examining their roles across different ICH tourism contexts.Finally, the fsQCA calibration anchors were distribution-driven rather than theory-based. Although alternative percentile anchors produced substantively consistent results, future studies should test theoretically grounded calibration criteria where available.

## 6 Conclusion

This study examined how ritualized experiences and perceived authenticity are associated with tourists’ willingness to support ICH inheritance in the context of Gansu Shehuo. By combining SEM and fsQCA, the study identified both the net associations of individual experiential dimensions and the configurational pathways associated with high and low willingness. The findings indicate that tourists’ willingness to support ICH inheritance is associated with different combinations of ritual distinctiveness, symbolic order, historical credibility, and lived authenticity.This study enriches the understanding of ICH tourism by linking tourists’ experiential perceptions with culturally oriented behavioural intentions and offers practical insights for the sustainable revitalization of living heritage through tourism.

## 7 Ethics statement

This study was reviewed and approved by the Institutional Review Board of the College of Tourism, Northwest Normal University on November 8, 2025. No numbered approval was issued for this study. The research protocol involved an on-site questionnaire survey conducted at the venues where Shehuo performances were staged.All participants were adults aged 18 years or older. Before participation, they received an informed consent form explaining the purpose of the study, the voluntary nature of participation, confidentiality measures, the academic use of the data, and their right to withdraw at any time.Written informed consent was obtained from all participants before they proceeded to complete the questionnaire. No personally identifiable information was collected, and all data were anonymized and stored confidentially.
