# Supplementary material for: Ritualized experiences and perceived authenticity in shaping tourists’ willingness to support intangible cultural heritage inheritance
Source: PLoS One. 2026 Sep 21;21(9):e0358987. doi: 10.1371/journal.pone.0358987 (PMC13592599; doi:10.1371/journal.pone.0358987)
Supplement: Supplementary file 1 [file pone.0358987.s001.pdf]

Title:

Ritualized experiences and perceived authenticity in shaping tourists' willingness to inherit intangible cultural heritage

Manuscript Number: PONE-D-26-28004

ABSTRACT

- Delete (ICH) from the abstract
- Delete (SEM) from the abstract
- Delete (fsQCA) from the abstract
- Conclusion and recommendations are not captured

Introduction

- The background is too long

Literature Review and Hypotheses Development

- Under culture, from 108 to 115, there is no references
- From 135 to 148 no references

Hypothesis

- This should be reduce to only two

2.2 Perceived authenticity and tourists' willingness to inherit culture

- From page 159 to 165 no references
- From 176 to 184, there is no references

3.2 Data collection

- 290 is not appropriate in method. Since that talks about findings

4.1 Reliability and validity

- This should be moved to 3.2

- Table 2 should be moved to 3.2

Discussion and conclusion

- Discussion should be separated from conclusion
- Conclusion should be brief

General comments

- The manuscript is very lengthy
- The authors should check the appropriate format for presenting manuscript
- Method section should explicitly address only that
- Results section should explain only results
- The tables are too long
