# Supplementary material for: Ritualized experiences and perceived authenticity in shaping tourists’ willingness to support intangible cultural heritage inheritance
Source: PLoS One. 2026 Sep 21;21(9):e0358987. doi: 10.1371/journal.pone.0358987 (PMC13592599; doi:10.1371/journal.pone.0358987)
Supplement: Supplementary file 2 [file pone.0358987.s003.docx]

Respond to Reviewers

Editor：

1. Please ensure that your manuscript meets PLOS ONE's style requirements, including those for file naming.

**Response:**Thank you for this reminder. We have carefully checked the revised manuscript against the PLOS ONE formatting requirements, including the main-text format, title and author information, section organization, figure and table presentation, reference style, and file naming. The revised submission files have been renamed and formatted accordingly.

2. Please provide additional details regarding participant consent. In the ethics statement in the Methods and online submission information, please ensure that you have specified (1) whether consent was informed and (2) what type you obtained (for instance, written or verbal, and if verbal, how it was documented and witnessed). If your study included minors, state whether you obtained consent from parents or guardians. If the need for consent was waived by the ethics committee, please include this information.

**Response:**Thank you for pointing out this inconsistency. We have carefully reviewed the funding information and corrected the grant details in the Funding Information section and the Financial Disclosure section of the submission system. The grant numbers and funding agency information are now consistent across all submission materials.

3. We note that the grant information you provided in the ‘Funding Information’ and ‘Financial Disclosure’ sections do not match.When you resubmit, please ensure that you provide the correct grant numbers for the awards you received for your study in the ‘Funding Information’ section.

**Response:**Thank you for pointing out this inconsistency. We have carefully reviewed the funding information and corrected the grant details in the Funding Information section and the Financial Disclosure section of the submission system. The grant numbers and funding agency information are now consistent across all submission materials.

4. Please provide a complete Data Availability Statement in the submission form, ensuring you include all necessary access information or a reason for why you are unable to make your data freely accessible. If your research concerns only data provided within your submission, please write "All data are in the manuscript and/or supporting information files" as your Data Availability Statement.

**Response:**Thank you for this important comment. We agree that a complete Data Availability Statement is necessary to improve the transparency and reproducibility of the study. In response, we will deposit the anonymized dataset underlying the findings of this study in a recognized public data repository before the final submission of the revised manuscript. The repository name, DOI, and permanent access link will be provided in the Data Availability Statement in both the manuscript and the online submission system accordingly.

We have also revised the Data Availability Statement to clarify the accessibility of the research data and will update it with the complete access information once the dataset has been deposited.

Reviewer #1:

1. The theoretical contribution needs to be more clearly articulated. While ritualized experiences and perceived authenticity are both well-established constructs in heritage tourism literature, the novelty of integrating these concepts and the specific contribution to ICH tourism and cultural sustainability remain insufficiently justified. The authors should clearly explain how their framework advances existing research.

**Response:**Thank you for this important and constructive comment. We agree that the original manuscript did not sufficiently explain the theoretical value of integrating ritualized experiences and perceived authenticity. In response, we have revised the Introduction and substantially rewritten Section 5.2. First, we clarify that the two constructs represent distinct but complementary mechanisms. Ritualized experiences explain how ICH meanings are enacted and communicated through symbolic procedures, ceremonial order, and participation, whereas perceived authenticity explains how tourists evaluate the credibility, meaning, and genuineness of these experiences. Their integration therefore connects the performative presentation of ICH with tourists’ interpretation and internalization of heritage meanings. Second, we explain why this framework is particularly relevant to ICH, which is continuously reproduced through performance, ritual practice, community participation, and situated interaction rather than preserved only as a material object. Third, we clarify that the study advances existing research by shifting the outcome from conventional market-oriented responses to tourists’ willingness to support ICH inheritance, thereby establishing a more direct link between tourism experience and cultural sustainability. Finally, the combined use of SEM and fsQCA reveals both heterogeneous net effects and alternative configurational pathways, providing a complexity-oriented explanation of how ritualized and authentic experiences jointly contribute to ICH inheritance.

2. Second, the concept of “tourists’ willingness to inherit culture” requires stronger conceptual clarification. The current measurement items include recommendations to others, participation intentions, and willingness to invest time and money, which appear to capture different dimensions of support behavior. The authors should define whether this construct represents cultural support, heritage stewardship, or actual inheritance intention and justify its relevance to tourists rather than local communities.

**Response:**Thank you for this important comment. We have retained the term “tourists’ willingness to inherit culture” because it follows the established conceptualization and measurement of Ruan et al. [17]. However, we have clarified that it represents a tourist-oriented intention to contribute to ICH continuity through communication, continued attention, participation, and the commitment of time or financial resources, rather than actual mastery of heritage skills or community-based stewardship. We also explain that the four items are related behavioral manifestations of the same underlying intention. Finally, the revised manuscript clarifies that tourists provide complementary support, while local communities and heritage bearers remain the primary agents of cultural practice and intergenerational transmission.

3. Third, the manuscript reports "N/A" under the ethics statement despite collecting questionnaire data from 390 tourists. The authors should provide details regarding ethical approval, informed consent procedures, and participant confidentiality in accordance with journal requirements. Similarly, the data availability statement currently contains placeholder text and should be replaced with a valid repository link and DOI.

**Response:**Thank you for this important comment. We agree that the ethics statement and data availability statement should be clearly reported. In response, we have replaced the original “N/A” ethics statement with a detailed ethics statement. The study has been reviewed and approved by the institutional review committee of the College of Tourism, Northwest Normal University. The committee confirmed that the research design and implementation plan were scientifically sound, fair, and would not cause harm or foreseeable risk to participants. We have also clarified that all participants were adults and were recruited based on voluntary participation and informed consent. Before completing the questionnaire, participants were informed of the research purpose, participation procedures, confidentiality measures, and their right to withdraw at any time. The questionnaire did not collect personally identifiable information, and all data were anonymized and used only for academic research.

In addition, the anonymized dataset underlying the findings of this study will be deposited in a public repository, and the Data Availability Statement will be updated with the repository name and permanent DOI or link before final submission.

4. Fourth, the methodology section requires further elaboration. The authors should provide more details regarding the scale adaptation process, pilot testing, translation and back-translation procedures, and report tests for common method bias, given that all variables were measured using self-reported questionnaires. In addition, the rationale for the fsQCA calibration anchors and threshold values should be more clearly justified.

**Response:**Thank you for this constructive comment. We agree that the original manuscript provided only a brief description of the scale adaptation and fsQCA procedures. We have therefore expanded Sections 3.2 and 3.4 and added the common method bias assessment to the Research Design section.

First, although the original manuscript mentioned translation, back-translation, expert review, and pilot testing, the revised Section 3.2 now provides further details about these procedures.Specifically, the original English items were translated into Chinese and independently back-translated into English, and differences between the original and back-translated versions were discussed to ensure semantic equivalence. Experts in tourism management from Gansu Province subsequently assessed the clarity, conceptual consistency, and contextual relevance of the items. A pilot survey was also conducted among tourists to identify ambiguous wording and comprehension difficulties, after which minor wording adjustments were made before the formal survey. We additionally clarified the procedural measures used to reduce potential common method bias, including respondent anonymity, confidentiality, and the use of concise and context-specific item wording.

Second, because all variables were measured using self-reported questionnaires, common method bias was statistically assessed before the reliability and validity analysis. Harman’s single-factor test extracted seven factors with eigenvalues greater than 1, and the first unrotated factor explained 35.21% of the total variance, below the 50% threshold. In addition, the single-factor measurement model showed a substantially poorer fit (χ²/df = 9.391, CFI = 0.544, TLI = 0.506, and RMSEA = 0.147) than the proposed eight-factor model (χ²/df = 1.100, CFI = 0.995, TLI = 0.994, and RMSEA = 0.016). These results indicate that common method bias was unlikely to seriously affect the findings.

Third, Section 3.4 has been revised to clarify the fsQCA calibration procedure and analytical thresholds.Based on the original distribution of each variable, the 95%, 50%, and 5% percentiles were set as the thresholds for full membership, the crossover point, and full non-membership, respectively. The condition and outcome variables were then transformed into fuzzy-set membership scores ranging from 0 to 1.

We further clarified that the consistency threshold for identifying a necessary condition was set at 0.90. For the truth-table analysis, the case frequency threshold was set at 1, the raw consistency threshold at 0.80, and the PRI consistency threshold at 0.75. These thresholds were used to retain empirically observed configurations while excluding contradictory configurations that simultaneously supported both the presence and absence of the outcome.

These revisions improve the transparency and reproducibility of the scale adaptation, data-quality assessment, and configurational analysis procedures.

5. Finally, the manuscript would benefit from careful language editing. Some terminology is used inconsistently throughout the paper, particularly terms such as "inherit culture," "support ICH inheritance," and "heritage continuity." Maintaining consistent terminology and improving sentence clarity would enhance the overall readability of the manuscript.

**Response:**Thank you for this valuable and constructive comment. We agree that the inconsistent use of terminology in the original manuscript may have caused conceptual ambiguity and affected the readability of the paper. In response, we carefully reviewed the manuscript and revised the terminology related to the focal construct. Specifically, because the present study focuses on Gansu Shehuo as a form of living intangible cultural heritage, we clarified that the focal construct refers to tourists’ willingness to inherit intangible cultural heritage in the context of ICH tourism. This construct was adapted from Ruan et al.’s (2024) concept of “tourists’ willingness to inherit culture” and contextualized to the specific setting of intangible cultural heritage tourism. We further clarified that this construct refers to tourists’ intention to contribute to ICH inheritance through cultural communication, continued attention, participation in inheritance-related activities, and the commitment of time or financial resources. It does not imply that tourists replace local communities or heritage bearers in mastering or intergenerationally transmitting heritage knowledge and skills. Rather, tourists play a complementary role in supporting ICH continuity. We also revised related expressions to distinguish the focal construct from broader process-oriented terms. In the revised manuscript, “support ICH inheritance” is used only to describe the behavioural content or supportive role associated with the construct, while “ICH continuity” and “sustainable ICH transmission” are used to describe the broader cultural process and long-term outcome. The abbreviation WIC has been retained in the figures and tables to remain consistent with the construct adapted from Ruan et al. (2024), but its meaning has been clarified in the context of intangible cultural heritage tourism. In addition, we improved sentence clarity and corrected several language, punctuation, and formatting issues, including hypothesis formatting and unclear expressions in the abstract, introduction, hypotheses, measurement section, and discussion.

Reviewer #2:

1. ABSTRACT
   • Delete (ICH) from the abstract
   • Delete (SEM) from the abstract
   • Delete (fsQCA) from the abstract
   • Conclusion and recommendations are not captured
   Introduction
   • The background is too long

**Response:**Thank you for this helpful comment. In response, we revised both the Abstract and the Introduction. In the Abstract, we removed the abbreviations “ICH,” “SEM,” and “fsQCA” and presented the relevant terms in full. We also added a clearer concluding statement to emphasize that the sustainable revitalization of intangible cultural heritage depends on the coordinated development of ritual distinctiveness, symbolic order, historical credibility, and lived authenticity. In addition, practical recommendations were incorporated by suggesting that heritage tourism managers preserve distinctive ritual procedures, strengthen historically credible interpretation, and create participatory experiences that enhance tourists’ emotional engagement and support for cultural continuity.

We also condensed the background of the Introduction. Specifically, we removed repetitive explanations concerning tourists’ heritage meaning-making and shortened the discussion of the relationship between ritualized experiences and perceived authenticity. The revised Introduction now moves more directly from the conceptual clarification of tourists’ willingness to inherit intangible cultural heritage to the research gap, empirical context, study objectives, and theoretical contributions.

2. Literature Review and Hypotheses Development
   • Under culture, from 108 to 115, there is no references
   • From 135 to 148 no references

**Response:**Thank you for pointing this out. We agree that the original manuscript did not provide sufficient references to support the relevant statements in the literature review. In response, we have added appropriate citations to strengthen the theoretical grounding of these paragraphs. Specifically, we supplemented references to support the discussion of how intangible cultural heritage is reproduced through performance, ritual practice, community participation, and situated interaction. We also added references to support the multidimensional nature of ritualized experiences and the ways in which these experiences are co-constructed by organizers, performers, local communities, and tourists. In addition, relevant literature has been added to substantiate the discussion of tourists’ perceptions of cultural distinctiveness, symbolic order, bodily participation, emotional involvement, and cultural significance in intangible cultural heritage tourism. These revisions improve the evidential support and academic rigor of the literature review.

3. Hypothesis
   • This should be reduce to only two

**Response:**Thank you for this helpful suggestion. We carefully considered the possibility of reducing the hypotheses to two. However, after re-examining the theoretical logic and analytical design of the study, we decided to retain the current three-part hypothesis structure. This is because the hypotheses correspond to three distinct but connected research purposes. The H1a–H1d hypotheses examine the net effects of the four dimensions of ritualized experiences on tourists’ willingness to inherit intangible cultural heritage, while the H2a–H2c hypotheses examine the net effects of the three dimensions of perceived authenticity on tourists’ willingness to inherit intangible cultural heritage. H3 serves a different purpose because it is grounded in complexity theory and focuses on the configurational combinations of ritualized experiences and perceived authenticity. Therefore, H3 does not simply repeat the dimension-specific hypotheses, but provides the theoretical basis for the fuzzy-set qualitative comparative analysis and for examining causal complexity, equifinality, and asymmetric relationships. Reducing the hypotheses to only two broad statements would obscure the distinction between the net-effect logic tested by structural equation modelling and the configurational logic tested by fuzzy-set qualitative comparative analysis. To address the reviewer’s concern, we have clarified the theoretical logic underlying the current hypothesis structure in the literature review.

4. 2.2 Perceived authenticity and tourists’ willingness to inherit culture
   • From page 159 to 165 no references
   • From 176 to 184, there is no references

**Response:**Thank you for pointing this out. We agree that the original manuscript did not provide sufficient references in these parts of Section 2.2. In response, we have added relevant citations to strengthen the theoretical support for the discussion of perceived authenticity. Specifically, we supplemented references on the multidimensional nature of perceived authenticity, including objective authenticity, constructive authenticity, and existential authenticity. We also added references to support the argument that perceived authenticity influences tourists’ evaluations of cultural credibility, symbolic meaning, emotional resonance, and subsequent heritage-related attitudes and behavioural intentions. These added references provide a stronger theoretical basis for the development of H2a–H2c, which concern the relationship between perceived authenticity and tourists’ willingness to inherit intangible cultural heritage.

5. 3.2 Data collection
   • 290 is not appropriate in method. Since that talks about findings

**Response:**Thank you for this helpful comment. We agree that the original sentence in Section 3.2 described sample characteristics and included an interpretive statement, which was not fully appropriate for the data collection section. In response, we revised this paragraph by retaining only the information related to the data collection procedure, questionnaire distribution, valid sample size, and response rate. The description of respondent characteristics has been presented more neutrally by referring readers to Table 1.

6. 4.1 Reliability and validity
   • This should be moved to 3.2
   • Table 2 should be moved to 3.2

**Response:**Thank you for this helpful comment. We agree that the reliability and validity assessment is more closely related to the methodological procedures than to the presentation of substantive empirical findings. In response, we relocated the common method bias assessment and the reliability and validity analysis from the original Results section to the Research Design section. Table 2 was moved together with the reliability and convergent validity assessment. Because Table 3 reports discriminant validity and correlations among the constructs, it was also relocated alongside Table 2 to present the measurement assessment coherently. These analyses and tables are now placed immediately after the data collection information and Table 1, thereby preserving the numerical order of the tables while clearly separating measurement assessment from the substantive results. As a result, the revised Results section now begins directly with the structural model results, and the subsequent subsections in Section 4 have been renumbered accordingly.
