# Supplementary material for: Ritualized experiences and perceived authenticity in shaping tourists’ willingness to support intangible cultural heritage inheritance
Source: PLoS One. 2026 Sep 21;21(9):e0358987. doi: 10.1371/journal.pone.0358987 (PMC13592599; doi:10.1371/journal.pone.0358987)
Supplement: Supplementary file 3 [file pone.0358987.s004.docx]

Respond to Reviewers

Editor：

1. If the reviewer comments include a recommendation to cite specific previously published works, please review and evaluate these publications to determine whether they are relevant and should be cited. There is no requirement to cite these works unless the editor has indicated otherwise.

**Response:**Thank you for this guidance. We have carefully reviewed the references suggested in the reviewer comments and evaluated their relevance to the present study. We have only retained or added references that are directly relevant to the theoretical and methodological development of the manuscript.

2. Please review your reference list to ensure that it is complete and correct. If you have cited papers that have been retracted, please include the rationale for doing so in the manuscript text, or remove these references and replace them with relevant current references. Any changes to the reference list should be mentioned in the rebuttal letter that accompanies your revised manuscript. If you need to cite a retracted article, indicate the article’s retracted status in the References list and also include a citation and full reference for the retraction notice.

**Response:**Thank you for this important reminder. We have carefully reviewed the reference list to ensure that all cited works are complete and correctly presented. We also checked the cited literature for retraction status and found no retracted articles that require special disclosure or replacement. Any reference additions or corrections made during the revision have been reflected in the revised manuscript and rebuttal letter.

Reviewer #1:

1. The dependent construct, “tourists’ willingness to inherit intangible cultural heritage,” is still conceptually awkward because its items capture recommendation, continued attention, participation, and willingness to contribute time or money rather than actual cultural inheritance; the authors should either justify this terminology more strongly or consider a clearer label such as willingness to support ICH continuity or heritage-support intention.

**Response:**Thank you for this important and insightful comment. We agree that the term “tourists’ willingness to inherit intangible cultural heritage” may imply that tourists themselves undertake the direct inheritance or intergenerational transmission of ICH, whereas the items used in this study primarily capture tourists’ intentions to recommend, continue paying attention to, participate in, and contribute time or financial resources to the continuation of ICH. To avoid this conceptual ambiguity, we have revised the terminology throughout the manuscript and now use “tourists’ willingness to support ICH inheritance” to describe the focal outcome construct.

This revised label more accurately reflects the behavioral intentions measured by the scale while retaining its theoretical connection to the original construct adapted from Ruan et al. [19]. We have also further clarified in the Introduction and Measurement Scales sections that this construct does not refer to tourists directly mastering, practicing, or intergenerationally transmitting heritage knowledge and skills. Rather, it represents tourists’ willingness to support ICH continuity through cultural communication, continued attention, participation in inheritance-related activities, and the contribution of time or financial resources. Local communities and heritage bearers remain the principal actors responsible for cultural practice and intergenerational transmission, while tourists may play a complementary supportive role.

Accordingly, we have revised the relevant terminology in the title, Abstract, Introduction, hypotheses, measurement description, Results, Discussion, Conclusion, tables, and figures to ensure conceptual clarity and terminological consistency throughout the manuscript. We believe that this revision more accurately reflects the content of the measurement items and avoids overstating tourists’ role in the actual inheritance of intangible cultural heritage.

2. The theoretical integration of ritualized experience and authenticity also needs further development because the current model treats them as parallel predictors, although ritualized experiences could plausibly shape authenticity perceptions.

**Response:**Thank you for this insightful comment. We agree that ritualized experiences may plausibly contribute to tourists’ authenticity perceptions, and that this potential relationship deserves clearer theoretical consideration.

In the revised manuscript, we have further clarified the rationale for treating ritualized experiences and perceived authenticity as two conceptually distinct but complementary antecedent domains. Ritualized experiences primarily capture the performative and structural characteristics of the tourism experience, including distinctiveness, commitment, ceremonial order, and non-functionality, whereas perceived authenticity reflects tourists’ cognitive and experiential evaluations of whether the heritage experience is historically credible, socially meaningful, and personally genuine. Accordingly, the present study focuses on their differentiated and configurational associations with tourists’ willingness to support ICH inheritance rather than assuming a specific causal ordering between the two constructs.

At the same time, we acknowledge that ritualized experiences may shape authenticity perceptions in some contexts. We have therefore added a discussion of this plausible sequential relationship in the theoretical development section and clarified that the present cross-sectional design does not allow us to establish whether ritualized experiences precede perceived authenticity. We have also identified this relationship as an important direction for future research, for example through longitudinal or mediation-based designs.

We believe that these revisions strengthen the theoretical integration of the two constructs while preserving the current study’s focus on their complementary net and configurational roles.

3. H3 should also be reformulated in set-theoretic terms because fsQCA identifies sufficient configurations rather than conventional “positive effects.”

**Response:**Thank you for this important comment. We agree that the original wording of H3 relied too heavily on conventional linear-effect terminology and did not adequately reflect the set-theoretic logic of fsQCA. In the revised manuscript, we have therefore reformulated H3 to emphasize sufficient configurations associated with both high and low levels of tourists’ willingness to support ICH inheritance.

Specifically, H3 has been revised as follows:Different configurations of ritualized experiences and perceived authenticity constitute sufficient pathways associated with high and low tourists’ willingness to support ICH inheritance, and the configurations associated with low willingness need not be the inverse of those associated with high willingness.

This revision better reflects the logic of fsQCA, which focuses on sufficient combinations of conditions rather than conventional independent positive or negative effects. It also aligns with the configurational principle of asymmetry, whereby the conditions associated with the presence of an outcome are not necessarily the mirror image of those associated with its absence.

4. The fsQCA procedure needs stronger justification and robustness assessment, particularly regarding the use of the 95th, 50th, and 5th percentile calibration anchors and the case-frequency threshold of 1 for a sample of 390. Please provide justification for these choices and conduct sensitivity analyses using alternative calibration anchors and higher frequency/consistency thresholds to demonstrate the robustness of the identified configurations.

**Response:**Thank you for this important comment. We agree that the calibration anchors and truth-table thresholds require clearer justification and that sensitivity analyses are necessary to assess the robustness of the configurational results.

In the revised manuscript, we have first clarified the rationale for the original calibration procedure. Because no externally established substantive calibration benchmarks were available for the constructs measured in this study, we adopted percentile-based direct calibration, using the 95th, 50th, and 5th percentiles as the thresholds for full membership, the crossover point, and full non-membership, respectively.

We further conducted sensitivity analyses using alternative calibration anchors and more stringent truth-table thresholds. Specifically, the calibration anchors were changed from the 95th, 50th, and 5th percentiles to the 90th, 50th, and 10th percentiles. In addition, the case-frequency threshold was increased from 1 to 3 and, in a more stringent specification, to 5, while the raw-consistency threshold was increased from 0.80 to 0.85, with the PRI-consistency threshold retained at 0.75.

The sensitivity analyses showed that the substantive configurational pattern remained stable under these alternative specifications. Increasing the case-frequency threshold mainly excluded low-frequency truth-table rows, while the configurations supported by larger numbers of cases remained. In particular, the low-willingness configuration characterized by the joint absence of the major experiential conditions remained stable under both the frequency-3 and frequency-5 specifications. The principal configurational structure associated with high willingness also remained substantively consistent, although some low-frequency combinations were excluded under the more stringent thresholds.

These additional analyses indicate that the main configurational conclusions are not driven solely by the original calibration anchors or truth-table thresholds. We have added the methodological justification and robustness results to the revised manuscript.

5. The contrast between the SEM and fsQCA results should be discussed more explicitly. In particular, commitment and constructive authenticity are not significant in the SEM model, yet they appear as relevant conditions in some configurational solutions. Please explain how these conditions can be configurationally important despite lacking significant independent net effects.

**Response:**Thank you for this important comment. We agree that the relationship between the SEM and fsQCA findings requires clearer explanation. In the revised manuscript, we have explicitly clarified that the two approaches address different analytical questions.

SEM estimates the independent net association of each predictor with tourists’ willingness to support ICH inheritance while accounting for the other predictors, whereas fsQCA examines whether conditions become relevant as part of sufficient configurations. Therefore, the nonsignificant SEM coefficients for commitment and constructive authenticity should not be interpreted as evidence that these conditions are unimportant. Rather, their presence in particular fsQCA solutions indicates configurational relevance: they may contribute to the outcome when combined with complementary ritualized-experience or authenticity conditions, even though they do not exhibit statistically significant independent net associations.

We have added this explanation to the Discussion section and further clarified in the Theoretical Implications section that the difference between the SEM and fsQCA results reflects the complementarity of net-effect and configurational perspectives rather than a contradiction between the two methods.

6. The causal language should be moderated throughout the manuscript. The cross-sectional and self-reported nature of the data does not establish temporal or causal relationships, and the term “causal asymmetry” in fsQCA should not be interpreted as experimental causality.

**Response:**Thank you for this important comment. We agree that the causal language in the manuscript should be moderated because the study is based on cross-sectional and self-reported data, which do not permit strong temporal or causal inference. In the revised manuscript, we have systematically replaced causal expressions such as “influence,” “effect,” “enhance,” “generate,” “foster,” “determine,” and “lead to” with more cautious terms such as “association,” “net association,” “configurational relevance,” and “configurations associated with the outcome,” where appropriate. We have also revised the SEM results, fsQCA interpretation, Discussion, Practical Implications, and Conclusion to ensure that the findings are presented as associations rather than causal effects.

We have further clarified the meaning of “causal asymmetry” in the fsQCA analysis. In the revised manuscript, this term is explicitly used in the set-theoretic sense, indicating that configurations associated with the presence of an outcome need not be the inverse of those associated with its absence; it does not imply experimental or temporal causality.

7. Please provide more information on the sampling strategy, the distribution of questionnaires across the study sites, the basis for determining the sample size, and the exact data-collection period. The timing should also be clearly consistent with the reported ethics approval date.

**Response:**Thank you for this important comment. We agree that the sampling and data-collection procedures required further clarification. In the revised manuscript, we have expanded Section 3.3 to provide additional information on the sampling strategy, sample distribution across the study sites, sample-size adequacy, and the exact data-collection period.

Specifically, we now clarify that data were collected from November 18, 2025 to January 8, 2026 using an on-site non-probability sampling strategy. A total of 425 questionnaires were collected, including 125 in Yongdeng County, 198 in Tongwei County, and 102 in Tianshui, of which 390 were retained as valid. We further added a sample-size adequacy assessment, noting that the final sample exceeded the commonly used ratio of 10 observations per measured item for SEM. The reported data-collection period also occurred after the ethics approval date of November 8, 2025. These revisions improve the transparency of the sampling and data-collection procedures.

8. The common method bias tests do not completely rule out the possibility of shared-method effects. Please interpret the results more cautiously and acknowledge the limitations of relying on single-source self-reported data.

**Response:**Thank you for this important comment. We agree that the statistical tests used to assess common method bias cannot completely rule out the possibility of shared-method effects. In the revised manuscript, we have therefore moderated our interpretation of the common method bias assessment.

Specifically, we revised the original statement to clarify that the results suggest that common method bias is unlikely to constitute a major threat to the findings, while the possibility of residual shared-method variance cannot be completely excluded. This more cautious wording avoids overstating the ability of Harman’s single-factor test and the single-factor measurement model to eliminate potential common method bias.We have also explicitly acknowledged the limitation of relying on single-source self-reported data in Section 5.4, Limitations and Future Research.

9. The outcome construct requires further justification because the four items capture somewhat heterogeneous forms of support, including recommendation, continued attention, participation, and the contribution of time or money. Please explain more clearly why these items can be treated as indicators of a single construct.

**Response:**Thank you for this important comment. We agree that the four outcome items capture different forms of heritage-supportive intention and therefore require clearer conceptual justification. In the revised manuscript, we have clarified that these items are not intended to represent identical behaviours, but rather complementary manifestations of a broader willingness to support ICH inheritance.

Specifically, recommendation and continued attention reflect communicative and attentional support, participation reflects engagement intention, and willingness to contribute time or financial resources reflects resource-based support. Despite these differences in form, all four items share a common underlying orientation toward supporting the continuity of ICH rather than actual heritage inheritance or intergenerational transmission. We have added this clarification to Section 3.2, Measurement Scales, to strengthen the conceptual basis for treating the four items as indicators of a single construct.

10. The practical implications should be framed as evidence-informed suggestions rather than as proven intervention effects. Please moderate prescriptive language and avoid implying that the proposed managerial actions will necessarily produce the desired outcomes.

**Response:**Thank you for this helpful comment. We agree that the practical implications should be presented as evidence-informed suggestions rather than as proven intervention effects. In the revised manuscript, we have moderated the prescriptive language throughout the Practical Implications section.

Specifically, expressions such as “should strengthen,” “should improve,” and “should adopt” have been revised to more cautious formulations such as “may consider,” “may benefit from,” and “greater attention may be given to.” We have also revised the concluding statements to avoid implying that the proposed managerial actions will necessarily produce specific outcomes. The revised section now presents these recommendations as practical implications informed by the observed associations and configurational patterns rather than as established causal interventions.

11. The low-willingness configuration should be interpreted cautiously until sensitivity analyses demonstrate its robustness.

**Response:**Thank you for this important comment. We agree that the low-willingness configuration should be interpreted cautiously. In the revised manuscript, we have moderated the wording used to describe the low-outcome solution and avoided presenting it as a definitive or causal explanation.

Specifically, we now state that the identified low-willingness configuration is associated with the concurrent absence of multiple key experiential conditions and should be interpreted cautiously because only one low-outcome configuration was identified. In addition, the newly added robustness assessment shows that this configuration remained stable under alternative analytical specifications, providing additional support for its robustness.

12. The limitations section should additionally acknowledge non-probability sampling, single-source self-report data, and possible heterogeneity across Shehuo locations.

**Response:** Thank you for this important comment. We agree that these methodological limitations should be made more explicit. In the revised manuscript, we have expanded the Limitations and Future Research section to acknowledge that the on-site non-probability sampling strategy may limit sample representativeness, that the use of single-source self-reported data may introduce response bias and residual shared-method effects, and that potential heterogeneity across the three Shehuo study locations was not explicitly examined. We have also suggested that future research employ more systematic sampling strategies, incorporate multiple data sources, and further examine cross-location heterogeneity.

13.A final round of language editing and formatting consistency checks is recommended throughout the manuscript.

**Response:**Thank you for this helpful suggestion. We have conducted a comprehensive language and formatting review of the revised manuscript. Specifically, we standardized the terminology used for the outcome construct as “tourists’willingness to support ICH inheritance,”corrected inconsistent abbreviations and residual wording, and standardized the formatting of section headings, figure and table captions, punctuation, spacing, and capitalization.

We also corrected grammatical and typographical errors and ensured greater consistency in English spelling and academic expression throughout the manuscript. These revisions were made across the Abstract, main text, tables, figure captions, and notes to improve the overall clarity and presentation of the manuscript.

Reviewer #3:

1.Ethics approval number. The manuscript names the approving body (College of Tourism, Northwest Normal University IRB) but does not provide a specific approval number. PLOS ONE's reporting guidelines ask authors to "include the approval number if one was obtained." Please either insert the approval number or explicitly state that no numbered approval was issued for this study.

**Response:**Thank you for this helpful suggestion. We have clarified the ethics statement in the revised manuscript. The study was reviewed and approved by the Institutional Review Board of the College of Tourism, Northwest Normal University on November 8, 2025. As no numbered approval was issued for this study, we have now explicitly stated this in the Ethics Statement.

2.fsQCA calibration anchors. The 95 %/5 % percentile anchors are distribution-driven rather than theory- or logic-driven, which remains debated in the QCA literature. The authors provide a justification, but it would strengthen the manuscript to note this choice as a limitation in the Discussion, or to flag that future replications should test robustness against theory-based anchor points.

**Response:**Thank you for this helpful suggestion. We have added a brief limitation regarding the percentile-based calibration strategy in Section 5.4. We now clarify that the calibration anchors used in the fsQCA are distribution-driven rather than theory-based. Although the robustness analyses using alternative percentile anchors yielded substantively consistent results, we also note that future studies should further examine the stability of the findings using theoretically grounded calibration criteria where available.

3.Distinction between net-effect and configurational logic. The retention of H3 is well defended. To pre-empt reader confusion, I suggest adding one explicit sentence in the Discussion clarifying that a non-significant net effect in the SEM does not imply the condition is unimportant in a configuration—that is, the two analytical lenses answer different questions. This would make the relationship between H1/H2 and H3 fully transparent.

**Response:**Thank you for this helpful suggestion. We have added an explicit clarification in the Discussion section to distinguish the analytical logic of SEM and fsQCA. Specifically, we now explain that SEM assesses the independent net association of each predictor with the outcome, whereas fsQCA examines the configurational relevance of conditions within sufficient combinations. We further clarify that a nonsignificant SEM coefficient does not imply that a condition is unimportant in a configurational solution.
