# Supplementary material for: Ritualized experiences and perceived authenticity in shaping tourists’ willingness to support intangible cultural heritage inheritance
Source: PLoS One. 2026 Sep 21;21(9):e0358987. doi: 10.1371/journal.pone.0358987 (PMC13592599; doi:10.1371/journal.pone.0358987)
Supplement: Peer review history

# Peer review: Decision Letter 0

**DOI:** 10.1371/journal.pone.0358987.r001

**Authors:** Bo Pu

16 Jul 2026

Dear Dr. Song,

Thank you for submitting your manuscript to PLOS ONE. After careful consideration, we feel that it has merit but does not fully meet PLOS ONE’s publication criteria as it currently stands. Therefore, we invite you to submit a revised version of the manuscript that addresses the points raised during the review process.

Please submit your revised manuscript by Sep 14 2026 11:59PM. If you will need more time than this to complete your revisions, please reply to this message or contact the journal office at plosone@plos.org. When you're ready to submit your revision, log on to https://www.editorialmanager.com/pone/ and select the 'Submissions Needing Revision' folder to locate your manuscript file.

- A letter that responds to each point raised by the academic editor and reviewer(s). You should upload this letter as a separate file labeled 'Response to Reviewers'.
- A marked-up copy of your manuscript that highlights changes made to the original version. You should upload this as a separate file labeled 'Revised Manuscript with Track Changes'.
- An unmarked version of your revised paper without tracked changes. You should upload this as a separate file labeled 'Manuscript'.

If you would like to make changes to your financial disclosure, please include your updated statement in your cover letter. Guidelines for resubmitting your figure files are available below the reviewer comments at the end of this letter.

If applicable, we recommend that you deposit your laboratory protocols in protocols.io to enhance the reproducibility of your results. Protocols.io assigns your protocol its own identifier (DOI) so that it can be cited independently in the future. For instructions see: https://journals.plos.org/plosone/s/submission-guidelines#loc-laboratory-protocols. Additionally, PLOS ONE offers an option for publishing peer-reviewed Lab Protocol articles, which describe protocols hosted on protocols.io. Read more information on sharing protocols at https://plos.org/protocols?utm_medium=editorial-email&utm_source=authorletters&utm_campaign=protocols.

As the corresponding author, your ORCID iD is verified in the submission system and will appear in the published article. PLOS supports the use of ORCID, and we encourage all coauthors to register for an ORCID iD and use it as well. Please encourage your coauthors to verify their ORCID iD within the submission system before final acceptance, as unverified ORCID iDs will not appear in the published article. *Only*  the individual author can complete the verification step; PLOS staff *cannot*  verify ORCID iDs on behalf of authors.

We look forward to receiving your revised manuscript.

Kind regards,

Bo Pu, Ph.D.

Academic Editor

PLOS One

Journal Requirements:

When submitting your revision, we need you to address these additional requirements.

1. Please ensure that your manuscript meets PLOS ONE's style requirements, including those for file naming. The PLOS ONE style templates can be found at

https://journals.plos.org/plosone/s/file?id=wjVg/PLOSOne_formatting_sample_main_body.pdf and

https://journals.plos.org/plosone/s/file?id=ba62/PLOSOne_formatting_sample_title_authors_affiliations.pdf

2. Please provide additional details regarding participant consent. In the ethics statement in the Methods and online submission information, please ensure that you have specified (1) whether consent was informed and (2) what type you obtained (for instance, written or verbal, and if verbal, how it was documented and witnessed). If your study included minors, state whether you obtained consent from parents or guardians. If the need for consent was waived by the ethics committee, please include this information.

If you are reporting a retrospective study of medical records or archived samples, please ensure that you have discussed whether all data were fully anonymized before you accessed them and/or whether the IRB or ethics committee waived the requirement for informed consent. If patients provided informed written consent to have data from their medical records used in research, please include this information.

3. We note that the grant information you provided in the ‘Funding Information’ and ‘Financial Disclosure’ sections do not match.

When you resubmit, please ensure that you provide the correct grant numbers for the awards you received for your study in the ‘Funding Information’ section.

4. Please provide a complete Data Availability Statement in the submission form, ensuring you include all necessary access information or a reason for why you are unable to make your data freely accessible. If your research concerns only data provided within your submission, please write "All data are in the manuscript and/or supporting information files" as your Data Availability Statement.

5. Please include captions for your Supporting Information files at the end of your manuscript, and update any in-text citations to match accordingly. Please see our Supporting Information guidelines for more information: http://journals.plos.org/plosone/s/supporting-information.

6. If the reviewer comments include a recommendation to cite specific previously published works, please review and evaluate these publications to determine whether they are relevant and should be cited. There is no requirement to cite these works unless the editor has indicated otherwise.

Additional Editor Comments:

Please revise this manuscript carefully according to the comments.

[Note: HTML markup is below. Please do not edit.]

Reviewers' comments:

Reviewer's Responses to Questions

**Comments to the Author**

1. Is the manuscript technically sound, and do the data support the conclusions?

Reviewer #1: No

Reviewer #2: Partly

**********

2. Has the statistical analysis been performed appropriately and rigorously?

Reviewer #1: No

Reviewer #2: Yes

**********

3. Have the authors made all data underlying the findings in their manuscript fully available?

The PLOS Data policy

Reviewer #1: No

Reviewer #2: Yes

**********

4. Is the manuscript presented in an intelligible fashion and written in standard English?

Reviewer #1: No

Reviewer #2: Yes

**********

Reviewer #1: The manuscript addresses an important and timely topic in the field of intangible cultural heritage (ICH) tourism by examining how ritualized experiences and perceived authenticity influence tourists' willingness to inherit culture. The integration of Structural Equation Modeling (SEM) and fuzzy-set Qualitative Comparative Analysis (fsQCA) is commendable and provides both symmetric and asymmetric perspectives on tourists' behavioral intentions. The study is also strengthened by the use of Gansu Shehuo as an empirical context, which represents a rich and meaningful example of living cultural heritage.

Despite these strengths, the manuscript requires substantial revision before it can be considered for publication.

First, the theoretical contribution needs to be more clearly articulated. While ritualized experiences and perceived authenticity are both well-established constructs in heritage tourism literature, the novelty of integrating these concepts and the specific contribution to ICH tourism and cultural sustainability remain insufficiently justified. The authors should clearly explain how their framework advances existing research.

Second, the concept of "tourists' willingness to inherit culture" requires stronger conceptual clarification. The current measurement items include recommendations to others, participation intentions, and willingness to invest time and money, which appear to capture different dimensions of support behavior. The authors should define whether this construct represents cultural support, heritage stewardship, or actual inheritance intention and justify its relevance to tourists rather than local communities.

Third, the manuscript reports "N/A" under the ethics statement despite collecting questionnaire data from 390 tourists. The authors should provide details regarding ethical approval, informed consent procedures, and participant confidentiality in accordance with journal requirements. Similarly, the data availability statement currently contains placeholder text and should be replaced with a valid repository link and DOI.

Fourth, the methodology section requires further elaboration. The authors should provide more details regarding the scale adaptation process, pilot testing, translation and back-translation procedures, and report tests for common method bias, given that all variables were measured using self-reported questionnaires. In addition, the rationale for the fsQCA calibration anchors and threshold values should be more clearly justified.

Finally, the manuscript would benefit from careful language editing. Some terminology is used inconsistently throughout the paper, particularly terms such as "inherit culture," "support ICH inheritance," and "heritage continuity." Maintaining consistent terminology and improving sentence clarity would enhance the overall readability of the manuscript.

Overall, the study has the potential to contribute meaningfully to the literature on intangible cultural heritage tourism and cultural sustainability. However, the above concerns should be adequately addressed to strengthen the theoretical foundation, methodological rigor, and presentation quality of the manuscript.

Reviewer #2: ABSTRACT

• Delete (ICH) from the abstract

• Delete (SEM) from the abstract

• Delete (fsQCA) from the abstract

• Conclusion and recommendations are not captured

Introduction

• The background is too long

Literature Review and Hypotheses Development

• Under culture, from 108 to 115, there is no references

• From 135 to 148 no references

Hypothesis

• This should be reduce to only two

2.2 Perceived authenticity and tourists’ willingness to inherit culture

• From page 159 to 165 no references

• From 176 to 184, there is no references

3.2 Data collection

• 290 is not appropriate in method. Since that talks about findings

4.1 Reliability and validity

• This should be moved to 3.2

• Table 2 should be moved to 3.2

Discussion and conclusion

• Discussion should be separated from conclusion

• Conclusion should be brief

General comments

• The manuscript is very lengthy

• The authors should check the appropriate format for presenting manuscript

• Method section should explicitly address only that

• Results section should explain only results

• The tables are too long

**********

what does this mean?). If published, this will include your full peer review and any attached files.

If you choose “no”, your identity will remain anonymous but your review may still be made public.

**Do you want your identity to be public for this peer review?**  For information about this choice, including consent withdrawal, please see our Privacy Policy

Reviewer #1: **Yes:** Dr.Shuvasree Banerjee

Reviewer #2: No

**********

[NOTE: If reviewer comments were submitted as an attachment file, they will be attached to this email and accessible via the submission site. Please log into your account, locate the manuscript record, and check for the action link "View Attachments". If this link does not appear, there are no attachment files.]

To ensure your figures meet our technical requirements, please review our figure guidelines: https://journals.plos.org/plosone/s/figures

You may also use PLOS’s free figure tool, NAAS, to help you prepare publication quality figures: https://journals.plos.org/plosone/s/figures#loc-tools-for-figure-preparation.

NAAS will assess whether your figures meet our technical requirements by comparing each figure against our figure specifications.

Attachment Submitted filename: review of manuscript retualized.pdf

# Author comment: Author response to Decision Letter 1

**DOI:** 10.1371/journal.pone.0358987.r002

19 Aug 2026

Editor：

1.Please ensure that your manuscript meets PLOS ONE's style requirements, including those for file naming.

Response:Thank you for this reminder. We have carefully checked the revised manuscript against the PLOS ONE formatting requirements, including the main-text format, title and author information, section organization, figure and table presentation, reference style, and file naming. The revised submission files have been renamed and formatted accordingly.

2.Please provide additional details regarding participant consent. In the ethics statement in the Methods and online submission information, please ensure that you have specified (1) whether consent was informed and (2) what type you obtained (for instance, written or verbal, and if verbal, how it was documented and witnessed). If your study included minors, state whether you obtained consent from parents or guardians. If the need for consent was waived by the ethics committee, please include this information.

Response:Thank you for pointing out this inconsistency. We have carefully reviewed the funding information and corrected the grant details in the Funding Information section and the Financial Disclosure section of the submission system. The grant numbers and funding agency information are now consistent across all submission materials.

3.We note that the grant information you provided in the ‘Funding Information’ and ‘Financial Disclosure’ sections do not match.When you resubmit, please ensure that you provide the correct grant numbers for the awards you received for your study in the ‘Funding Information’ section.

Response:Thank you for pointing out this inconsistency. We have carefully reviewed the funding information and corrected the grant details in the Funding Information section and the Financial Disclosure section of the submission system. The grant numbers and funding agency information are now consistent across all submission materials.

4.Please provide a complete Data Availability Statement in the submission form, ensuring you include all necessary access information or a reason for why you are unable to make your data freely accessible. If your research concerns only data provided within your submission, please write "All data are in the manuscript and/or supporting information files" as your Data Availability Statement.

Response:Thank you for this important comment. We agree that a complete Data Availability Statement is necessary to improve the transparency and reproducibility of the study. In response, we will deposit the anonymized dataset underlying the findings of this study in a recognized public data repository before the final submission of the revised manuscript. The repository name, DOI, and permanent access link will be provided in the Data Availability Statement in both the manuscript and the online submission system accordingly.

We have also revised the Data Availability Statement to clarify the accessibility of the research data and will update it with the complete access information once the dataset has been deposited.

Reviewer #1:

1.The theoretical contribution needs to be more clearly articulated. While ritualized experiences and perceived authenticity are both well-established constructs in heritage tourism literature, the novelty of integrating these concepts and the specific contribution to ICH tourism and cultural sustainability remain insufficiently justified. The authors should clearly explain how their framework advances existing research.

Response:Thank you for this important and constructive comment. We agree that the original manuscript did not sufficiently explain the theoretical value of integrating ritualized experiences and perceived authenticity. In response, we have revised the Introduction and substantially rewritten Section 5.2. First, we clarify that the two constructs represent distinct but complementary mechanisms. Ritualized experiences explain how ICH meanings are enacted and communicated through symbolic procedures, ceremonial order, and participation, whereas perceived authenticity explains how tourists evaluate the credibility, meaning, and genuineness of these experiences. Their integration therefore connects the performative presentation of ICH with tourists’ interpretation and internalization of heritage meanings. Second, we explain why this framework is particularly relevant to ICH, which is continuously reproduced through performance, ritual practice, community participation, and situated interaction rather than preserved only as a material object. Third, we clarify that the study advances existing research by shifting the outcome from conventional market-oriented responses to tourists’ willingness to support ICH inheritance, thereby establishing a more direct link between tourism experience and cultural sustainability. Finally, the combined use of SEM and fsQCA reveals both heterogeneous net effects and alternative configurational pathways, providing a complexity-oriented explanation of how ritualized and authentic experiences jointly contribute to ICH inheritance.

2.Second, the concept of “tourists’ willingness to inherit culture” requires stronger conceptual clarification. The current measurement items include recommendations to others, participation intentions, and willingness to invest time and money, which appear to capture different dimensions of support behavior. The authors should define whether this construct represents cultural support, heritage stewardship, or actual inheritance intention and justify its relevance to tourists rather than local communities.

Response:Thank you for this important comment. We have retained the term “tourists’ willingness to inherit culture” because it follows the established conceptualization and measurement of Ruan et al. [17]. However, we have clarified that it represents a tourist-oriented intention to contribute to ICH continuity through communication, continued attention, participation, and the commitment of time or financial resources, rather than actual mastery of heritage skills or community-based stewardship. We also explain that the four items are related behavioral manifestations of the same underlying intention. Finally, the revised manuscript clarifies that tourists provide complementary support, while local communities and heritage bearers remain the primary agents of cultural practice and intergenerational transmission.

3.Third, the manuscript reports "N/A" under the ethics statement despite collecting questionnaire data from 390 tourists. The authors should provide details regarding ethical approval, informed consent procedures, and participant confidentiality in accordance with journal requirements. Similarly, the data availability statement currently contains placeholder text and should be replaced with a valid repository link and DOI.

Response:Thank you for this important comment. We agree that the ethics statement and data availability statement should be clearly reported. In response, we have replaced the original “N/A” ethics statement with a detailed ethics statement. The study has been reviewed and approved by the institutional review committee of the College of Tourism, Northwest Normal University. The committee confirmed that the research design and implementation plan were scientifically sound, fair, and would not cause harm or foreseeable risk to participants. We have also clarified that all participants were adults and were recruited based on voluntary participation and informed consent. Before completing the questionnaire, participants were informed of the research purpose, participation procedures, confidentiality measures, and their right to withdraw at any time. The questionnaire did not collect personally identifiable information, and all data were anonymized and used only for academic research.

In addition, the anonymized dataset underlying the findings of this study will be deposited in a public repository, and the Data Availability Statement will be updated with the repository name and permanent DOI or link before final submission.

4.Fourth, the methodology section requires further elaboration. The authors should provide more details regarding the scale adaptation process, pilot testing, translation and back-translation procedures, and report tests for common method bias, given that all variables were measured using self-reported questionnaires. In addition, the rationale for the fsQCA calibration anchors and threshold values should be more clearly justified.

Response:Thank you for this constructive comment. We agree that the original manuscript provided only a brief description of the scale adaptation and fsQCA procedures. We have therefore expanded Sections 3.2 and 3.4 and added the common method bias assessment to the Research Design section.

First, although the original manuscript mentioned translation, back-translation, expert review, and pilot testing, the revised Section 3.2 now provides further details about these procedures.Specifically, the original English items were translated into Chinese and independently back-translated into English, and differences between the original and back-translated versions were discussed to ensure semantic equivalence. Experts in tourism management from Gansu Province subsequently assessed the clarity, conceptual consistency, and contextual relevance of the items. A pilot survey was also conducted among tourists to identify ambiguous wording and comprehension difficulties, after which minor wording adjustments were made before the formal survey. We additionally clarified the procedural measures used to reduce potential common method bias, including respondent anonymity, confidentiality, and the use of concise and context-specific item wording.

Second, because all variables were measured using self-reported questionnaires, common method bias was statistically assessed before the reliability and validity analysis. Harman’s single-factor test extracted seven factors with eigenvalues greater than 1, and the first unrotated factor explained 35.21% of the total variance, below the 50% threshold. In addition, the single-factor measurement model showed a substantially poorer fit (χ²/df = 9.391, CFI = 0.544, TLI = 0.506, and RMSEA = 0.147) than the proposed eight-factor model (χ²/df = 1.100, CFI = 0.995, TLI = 0.994, and RMSEA = 0.016). These results indicate that common method bias was unlikely to seriously affect the findings.

Third, Section 3.4 has been revised to clarify the fsQCA calibration procedure and analytical thresholds.Based on the original distribution of each variable, the 95%, 50%, and 5% percentiles were set as the thresholds for full membership, the crossover point, and full non-membership, respectively. The condition and outcome variables were then transformed into fuzzy-set membership scores ranging from 0 to 1.

We further clarified that the consistency threshold for identifying a necessary condition was set at 0.90. For the truth-table analysis, the case frequency threshold was set at 1, the raw consistency threshold at 0.80, and the PRI consistency threshold at 0.75. These thresholds were used to retain empirically observed configurations while excluding contradictory configurations that simultaneously supported both the presence and absence of the outcome.

These revisions improve the transparency and reproducibility of the scale adaptation, data-quality assessment, and configurational analysis procedures.

5.Finally, the manuscript would benefit from careful language editing. Some terminology is used inconsistently throughout the paper, particularly terms such as "inherit culture," "support ICH inheritance," and "heritage continuity." Maintaining consistent terminology and improving sentence clarity would enhance the overall readability of the manuscript.

Response:Thank you for this valuable and constructive comment. We agree that the inconsistent use of terminology in the original manuscript may have caused conceptual ambiguity and affected the readability of the paper. In response, we carefully reviewed the manuscript and revised the terminology related to the focal construct. Specifically, because the present study focuses on Gansu Shehuo as a form of living intangible cultural heritage, we clarified that the focal construct refers to tourists’ willingness to inherit intangible cultural heritage in the context of ICH tourism. This construct was adapted from Ruan et al.’s (2024) concept of “tourists’ willingness to inherit culture” and contextualized to the specific setting of intangible cultural heritage tourism. We further clarified that this construct refers to tourists’ intention to contribute to ICH inheritance through cultural communication, continued attention, participation in inheritance-related activities, and the commitment of time or financial resources. It does not imply that tourists replace local communities or heritage bearers in mastering or intergenerationally transmitting heritage knowledge and skills. Rather, tourists play a complementary role in supporting ICH continuity. We also revised related expressions to distinguish the focal construct from broader process-oriented terms. In the revised manuscript, “support ICH inheritance” is used only to describe the behavioural content or supportive role associated with the construct, while “ICH continuity” and “sustainable ICH transmission” are used to describe the broader cultural process and long-term outcome. The abbreviation WIC has been retained in the figures and tables to remain consistent with the construct adapted from Ruan et al. (2024), but its meaning has been clarified in the context of intangible cultural heritage tourism. In addition, we improved sentence clarity and corrected several language, punctuation, and formatting issues, including hypothesis formatting and unclear expressions in the abstract, introduction, hypotheses, measurement section, and discussion.

Reviewer #2:

1.ABSTRACT

• Delete (ICH) from the abstract

• Delete (SEM) from the abstract

• Delete (fsQCA) from the abstract

• Conclusion and recommendations are not captured

Introduction

• The background is too long

Response:Thank you for this helpful comment. In response, we revised both the Abstract and the Introduction. In the Abstract, we removed the abbreviations “ICH,” “SEM,” and “fsQCA” and presented the relevant terms in full. We also added a clearer concluding statement to emphasize that the sustainable revitalization of intangible cultural heritage depends on the coordinated development of ritual distinctiveness, symbolic order, historical credibility, and lived authenticity. In addition, practical recommendations were incorporated by suggesting that heritage tourism managers preserve distinctive ritual procedures, strengthen historically credible interpretation, and create participatory experiences that enhance tourists’ emotional engagement and support for cultural continuity.

We also condensed the background of the Introduction. Specifically, we removed repetitive explanations concerning tourists’ heritage meaning-making and shortened the discussion of the relationship between ritualized experiences and perceived authenticity. The revised Introduction now moves more directly from the conceptual clarification of tourists’ willingness to inherit intangible cultural heritage to the research gap, empirical context, study objectives, and theoretical contributions.

2.Literature Review and Hypotheses Development

• Under culture, from 108 to 115, there is no references

• From 135 to 148 no references

Response:Thank you for pointing this out. We agree that the original manuscript did not provide sufficient references to support the relevant statements in the literature review. In response, we have added appropriate citations to strengthen the theoretical grounding of these paragraphs. Specifically, we supplemented references to support the discussion of how intangible cultural heritage is reproduced through performance, ritual practice, community participation, and situated interaction. We also added references to support the multidimensional nature of ritualized experiences and the ways in which these experiences are co-constructed by organizers, performers, local communities, and tourists. In addition, relevant literature has been added to substantiate the discussion of tourists’ perceptions of cultural distinctiveness, symbolic order, bodily participation, emotional involvement, and cultural significance in intangible cultural heritage tourism. These revisions improve the evidential support and academic rigor of the literature review.

3.Hypothesis

• This should be reduce to only two

Response:Thank you for this helpful suggestion. We carefully considered the possibility of reducing the hypotheses to two. However, after re-examining the theoretical logic and analytical design of the study, we decided to retain the current three-part hypothesis structure. This is because the hypotheses correspond to three distinct but connected research purposes. The H1a–H1d hypotheses examine the net effects of the four dimensions of ritualized experiences on tourists’ willingness to inherit intangible cultural heritage, while the H2a–H2c hypotheses examine the net effects of the three dimensions of perceived authenticity on tourists’ willingness to inherit intangible cultural heritage. H3 serves a different purpose because it is grounded in complexity theory and focuses on the configurational combinations of ritualized experiences and perceived authenticity. Therefore, H3 does not simply repeat the dimension-specific hypotheses, but provides the theoretical basis for the fuzzy-set qualitative comparative analysis and for examining causal complexity, equifinality, and asymmetric relationships. Reducing the hypotheses to only two broad statements would obscure the distinction between the net-effect logic tested by structural equation modelling and the configurational logic tested by fuzzy-set qualitative comparative analysis. To address the reviewer’s concern, we have clarified the theoretical logic underlying the current hypothesis structure in the literature review.

4.2.2 Perceived authenticity and tourists’ willingness to inherit culture

• From page 159 to 165 no references

• From 176 to 184, there is no references

Response:Thank you for pointing this out. We agree that the original manuscript did not provide sufficient references in these parts of Section 2.2. In response, we have added relevant citations to strengthen the theoretical support for the discussion of perceived authenticity. Specifically, we supplemented references on the multidimensional nature of perceived authenticity, including objective authenticity, constructive authenticity, and existential authenticity. We also added references to support the argument that perceived authenticity influences tourists’ evaluations of cultural credibility, symbolic meaning, emotional resonance, and subsequent heritage-related attitudes and behavioural intentions. These added references provide a stronger theoretical basis for the development of H2a–H2c, which concern the relationship between perceived authenticity and tourists’ willingness to inherit intangible cultural heritage.

5.3.2 Data collection

• 290 is not appropriate in method. Since that talks about findings

Response:Thank you for this helpful comment. We agree that the original sentence in Section 3.2 described sample characteristics and included an interpretive statement, which was not fully appropriate for the data collection section. In response, we revised this paragraph by retaining only the information related to the data collection procedure, questionnaire distribution, valid sample size, and response rate. The description of respondent characteristics has been presented more neutrally by referring readers to Table 1.

6.4.1 Reliability and validity

• This should be moved to 3.2

• Table 2 should be moved to 3.2

Response:Thank you for this helpful comment. We agree that the reliability and validity assessment is more closely related to the methodological procedures than to the presentation of substantive empirical findings. In response, we relocated the common method bias assessment and the reliability and validity analysis from the original Results section to the Research Design section. Table 2 was moved together with the reliability and convergent validity assessment. Because Table 3 reports discriminant validity and correlations among the constructs, it was also relocated alongside Table 2 to present the measurement assessment coherently. These analyses and tables are now placed immediately after the data collection information and Table 1, thereby preserving the numerical order of the tables while clearly separating measurement assessment from the substantive results. As a result, the revised Results section now begins directly with the structural model results, and the subsequent subsections in Section 4 have been renumbered accordingly.

Attachment Submitted filename: renamed_4e4cd.docx

# Peer review: Decision Letter 1

**DOI:** 10.1371/journal.pone.0358987.r003

**Authors:** Bo Pu

6 Sep 2026

Dear Dr. Song,

Thank you for submitting your manuscript to PLOS One. After careful consideration, we feel that it has merit but does not fully meet PLOS One’s publication criteria as it currently stands. Therefore, we invite you to submit a revised version of the manuscript that addresses the points raised during the review process.

Please submit your revised manuscript by Oct 06 2026 11:59PM. If you will need more time than this to complete your revisions, please reply to this message or contact the journal office at plosone@plos.org. When you're ready to submit your revision, log on to https://www.editorialmanager.com/pone/ and select the 'Submissions Needing Revision' folder to locate your manuscript file.

- A letter that responds to each point raised by the academic editor and reviewer(s). You should upload this letter as a separate file labeled 'Response to Reviewers'.
- A marked-up copy of your manuscript that highlights changes made to the original version. You should upload this as a separate file labeled 'Revised Manuscript with Track Changes'.
- An unmarked version of your revised paper without tracked changes. You should upload this as a separate file labeled 'Manuscript'.

If you would like to make changes to your financial disclosure, please include your updated statement in your cover letter. Guidelines for resubmitting your figure files are available below the reviewer comments at the end of this letter.

If applicable, we recommend that you deposit your laboratory protocols in protocols.io to enhance the reproducibility of your results. Protocols.io assigns your protocol its own identifier (DOI) so that it can be cited independently in the future. For instructions see: https://journals.plos.org/plosone/s/submission-guidelines#loc-laboratory-protocols. Additionally, PLOS One offers an option for publishing peer-reviewed Lab Protocol articles, which describe protocols hosted on protocols.io. Read more information on sharing protocols at https://plos.org/protocols?utm_medium=editorial-email&utm_source=authorletters&utm_campaign=protocols.

As the corresponding author, your ORCID iD is verified in the submission system and will appear in the published article. PLOS supports the use of ORCID, and we encourage all coauthors to register for an ORCID iD and use it as well. Please encourage your coauthors to verify their ORCID iD within the submission system before final acceptance, as unverified ORCID iDs will not appear in the published article. *Only*  the individual author can complete the verification step; PLOS staff *cannot*  verify ORCID iDs on behalf of authors.

We look forward to receiving your revised manuscript.

Kind regards,

Bo Pu, Ph.D.

Academic Editor

PLOS One

Journal Requirements:

If the reviewer comments include a recommendation to cite specific previously published works, please review and evaluate these publications to determine whether they are relevant and should be cited. There is no requirement to cite these works unless the editor has indicated otherwise.

Please review your reference list to ensure that it is complete and correct. If you have cited papers that have been retracted, please include the rationale for doing so in the manuscript text, or remove these references and replace them with relevant current references. Any changes to the reference list should be mentioned in the rebuttal letter that accompanies your revised manuscript. If you need to cite a retracted article, indicate the article’s retracted status in the References list and also include a citation and full reference for the retraction notice.

Additional Editor Comments (if provided):

please revise this manuscript according to the comments

[Note: HTML markup is below. Please do not edit.]

Reviewers' comments:

Reviewer's Responses to Questions

**Comments to the Author**

Reviewer #1: All comments have been addressed

Reviewer #3: (No Response)

**********

2. Is the manuscript technically sound, and do the data support the conclusions?

Reviewer #1: Partly

Reviewer #3: Yes

**********

3. Has the statistical analysis been performed appropriately and rigorously?

Reviewer #1: Yes

Reviewer #3: Yes

**********

4. Have the authors made all data underlying the findings in their manuscript fully available?

The PLOS Data policy

Reviewer #1: Yes

Reviewer #3: Yes

**********

5. Is the manuscript presented in an intelligible fashion and written in standard English?

Reviewer #1: (No Response)

Reviewer #3: Yes

**********

Reviewer #1: The manuscript addresses an important and timely topic in intangible cultural heritage tourism by examining how ritualized experiences and perceived authenticity shape tourists’ willingness to support cultural inheritance in the context of Gansu Shehuo. The integration of four dimensions of ritualized experience—uniqueness, commitment, ceremoniality, and non-functionality—with three dimensions of perceived authenticity—objective, constructive, and existential authenticity—is conceptually interesting, and the combined use of SEM and fsQCA provides a useful opportunity to compare net effects with configurational pathways. The revised manuscript is stronger than the earlier version, particularly because the authors have clarified the focal construct, expanded the scale adaptation and translation procedures, added common-method-bias assessment, improved the ethics statement, and strengthened the methodological explanation. However, several issues remain. The dependent construct, “tourists’ willingness to inherit intangible cultural heritage,” is still conceptually awkward because its items capture recommendation, continued attention, participation, and willingness to contribute time or money rather than actual cultural inheritance; the authors should either justify this terminology more strongly or consider a clearer label such as willingness to support ICH continuity or heritage-support intention. The theoretical integration of ritualized experience and authenticity also needs further development because the current model treats them as parallel predictors, although ritualized experiences could plausibly shape authenticity perceptions. H3 should also be reformulated in set-theoretic terms because fsQCA identifies sufficient configurations rather than conventional “positive effects.” The fsQCA procedure needs stronger justification and robustness testing, particularly because the study uses percentile-based calibration at the 95th, 50th, and 5th percentiles and a case-frequency threshold of only one despite a sample of 390; alternative calibration anchors and higher frequency/consistency thresholds should be tested to demonstrate that the six high-willingness configurations and one low-willingness configuration are stable. The contrast between the SEM and fsQCA results deserves more attention, especially because commitment and constructive authenticity are not significant in the SEM model yet appear as important conditions in some configurational solutions; this is one of the manuscript’s most interesting findings and should be explained more clearly as evidence that a condition can be configurationally relevant despite having no significant independent net effect. The authors should also moderate causal language because the cross-sectional self-report design does not establish temporal or causal relationships, and “causal asymmetry” in fsQCA should not be interpreted as experimental causality. More details are needed concerning sampling strategy, respondent distribution across the three study locations, and sample-size determination, and the exact period of data collection should be reported so that readers can verify that fieldwork occurred after the ethics approval dated 8 November 2025. The common-method-bias tests provide useful reassurance but do not completely eliminate the possibility of shared-method effects, so the conclusion should remain cautious. The measurement of the outcome construct also requires additional justification because recommendation, participation, continued attention, and financial/time commitment may represent heterogeneous behaviors, even if they load on a single factor. The practical implications should be framed as evidence-informed suggestions rather than proven intervention effects, and the low-willingness configuration should be interpreted cautiously until sensitivity analyses demonstrate its robustness. Finally, the limitations section should additionally acknowledge non-probability sampling, single-source self-report data, and possible heterogeneity across Shehuo locations, and the manuscript would benefit from a final language and formatting edit to ensure consistent terminology and presentation. Overall, the manuscript has a promising core and potentially publishable contribution, but these conceptual, methodological, and reporting issues should be addressed through a Major Revision.

Reviewer #3: 1. Response to Prior Review Comments (Round 1 Assessment)

I have checked the revised manuscript against the authors' point-by-point response and verified that the revisions are incorporated in the text itself, not merely in the response letter. My assessment of each prior comment is as follows:

(1) Missing citations in several passages. Citations have been added (performance, ritual, community participation, multidimensional authenticity). The reference list has been substantially expanded. Resolved.

(2) Hypotheses should be reduced to two. The authors retained H1a–d, H2a–c, and H3, defending this on the grounds that SEM tests net effects whereas fsQCA tests configurational pathways (distinct analytical logics). This is defensible and acceptable (see Minor Point 3 below).

(3) Exploratory phrasing in the data-collection section. Rephrased neutrally and cross-referenced to Table 1. Resolved.

(4) Reliability/validity placed in Results rather than Methods. Moved to the Research Design section; Tables 2–3 relocated accordingly. Resolved.

(5) Ethics statement was listed as "N/A" despite 390 questionnaires. Added IRB approval (College of Tourism, Northwest Normal University), voluntary adult participation, informed consent, and anonymity. Resolved.

(6) Data availability was a placeholder. Deposited at Figshare (DOI: 10.6084/m9.figshare.33150737). The DOI is present and valid. Resolved.

(7) Inconsistent funding number. Corrected to Grant No. 24YFGA047, Gansu Provincial Department of Science and Technology. The number is now consistent across sections. Resolved.

(8) fsQCA calibration anchors/thresholds under-justified. Added 95 %/50 %/5 % percentile anchors; necessity consistency 0.90; truth-table frequency 1, raw consistency 0.80, PRI 0.75. Resolved.

(9) "Willingness to inherit" applicability to tourists rather than local communities. Clarified as tourist-oriented inheritance-contribution intention (dissemination, attention, participation, investment), following Ruan et al. The conceptualisation is defensible. Resolved.

(10) Overly long background. Condensed abstract and introduction, removed unnecessary abbreviations, and added a concluding sentence. Resolved.

(11) Style, filename, and terminology consistency. Addressed throughout. Resolved.

All mandatory revisions have been satisfactorily resolved. The revision is of good quality, and the manuscript is suitable for publication.

2. Minor Points for the Final Proofs (Non-blocking)

The following are suggestions for clarification that can be handled at the proof stage; they do not require a further round of review.

Minor Point 1 — Ethics approval number. The manuscript names the approving body (College of Tourism, Northwest Normal University IRB) but does not provide a specific approval number. PLOS ONE's reporting guidelines ask authors to "include the approval number if one was obtained." Please either insert the approval number or explicitly state that no numbered approval was issued for this study.

Minor Point 2 — fsQCA calibration anchors. The 95 %/5 % percentile anchors are distribution-driven rather than theory- or logic-driven, which remains debated in the QCA literature. The authors provide a justification, but it would strengthen the manuscript to note this choice as a limitation in the Discussion, or to flag that future replications should test robustness against theory-based anchor points.

Minor Point 3 — Distinction between net-effect and configurational logic. The retention of H3 is well defended. To pre-empt reader confusion, I suggest adding one explicit sentence in the Discussion clarifying that a non-significant net effect in the SEM does not imply the condition is unimportant in a configuration—that is, the two analytical lenses answer different questions. This would make the relationship between H1/H2 and H3 fully transparent.

These three points do not diminish the contribution of the study.

**********

what does this mean?). If published, this will include your full peer review and any attached files.

If you choose “no”, your identity will remain anonymous but your review may still be made public.

**Do you want your identity to be public for this peer review?**  For information about this choice, including consent withdrawal, please see our Privacy Policy

Reviewer #1: **Yes:** Dr.Shuvasree Banerjee

Reviewer #3: No

**********

[NOTE: If reviewer comments were submitted as an attachment file, they will be attached to this email and accessible via the submission site. Please log into your account, locate the manuscript record, and check for the action link "View Attachments". If this link does not appear, there are no attachment files.]

To ensure your figures meet our technical requirements, please review our figure guidelines: https://journals.plos.org/plosone/s/figures

You may also use PLOS’s free figure tool, NAAS, to help you prepare publication quality figures: https://journals.plos.org/plosone/s/figures#loc-tools-for-figure-preparation.

NAAS will assess whether your figures meet our technical requirements by comparing each figure against our figure specifications.

# Author comment: Author response to Decision Letter 2

**DOI:** 10.1371/journal.pone.0358987.r004

7 Sep 2026

Respond to Reviewers

Editor：

1.If the reviewer comments include a recommendation to cite specific previously published works, please review and evaluate these publications to determine whether they are relevant and should be cited. There is no requirement to cite these works unless the editor has indicated otherwise.

Response:Thank you for this guidance. We have carefully reviewed the references suggested in the reviewer comments and evaluated their relevance to the present study. We have only retained or added references that are directly relevant to the theoretical and methodological development of the manuscript.

2.Please review your reference list to ensure that it is complete and correct. If you have cited papers that have been retracted, please include the rationale for doing so in the manuscript text, or remove these references and replace them with relevant current references. Any changes to the reference list should be mentioned in the rebuttal letter that accompanies your revised manuscript. If you need to cite a retracted article, indicate the article’s retracted status in the References list and also include a citation and full reference for the retraction notice.

Response:Thank you for this important reminder. We have carefully reviewed the reference list to ensure that all cited works are complete and correctly presented. We also checked the cited literature for retraction status and found no retracted articles that require special disclosure or replacement. Any reference additions or corrections made during the revision have been reflected in the revised manuscript and rebuttal letter.

Reviewer #1:

1.The dependent construct, “tourists’ willingness to inherit intangible cultural heritage,” is still conceptually awkward because its items capture recommendation, continued attention, participation, and willingness to contribute time or money rather than actual cultural inheritance; the authors should either justify this terminology more strongly or consider a clearer label such as willingness to support ICH continuity or heritage-support intention.

Response:Thank you for this important and insightful comment. We agree that the term “tourists’ willingness to inherit intangible cultural heritage” may imply that tourists themselves undertake the direct inheritance or intergenerational transmission of ICH, whereas the items used in this study primarily capture tourists’ intentions to recommend, continue paying attention to, participate in, and contribute time or financial resources to the continuation of ICH. To avoid this conceptual ambiguity, we have revised the terminology throughout the manuscript and now use “tourists’ willingness to support ICH inheritance” to describe the focal outcome construct.

This revised label more accurately reflects the behavioral intentions measured by the scale while retaining its theoretical connection to the original construct adapted from Ruan et al. [19]. We have also further clarified in the Introduction and Measurement Scales sections that this construct does not refer to tourists directly mastering, practicing, or intergenerationally transmitting heritage knowledge and skills. Rather, it represents tourists’ willingness to support ICH continuity through cultural communication, continued attention, participation in inheritance-related activities, and the contribution of time or financial resources. Local communities and heritage bearers remain the principal actors responsible for cultural practice and intergenerational transmission, while tourists may play a complementary supportive role.

Accordingly, we have revised the relevant terminology in the title, Abstract, Introduction, hypotheses, measurement description, Results, Discussion, Conclusion, tables, and figures to ensure conceptual clarity and terminological consistency throughout the manuscript. We believe that this revision more accurately reflects the content of the measurement items and avoids overstating tourists’ role in the actual inheritance of intangible cultural heritage.

2.The theoretical integration of ritualized experience and authenticity also needs further development because the current model treats them as parallel predictors, although ritualized experiences could plausibly shape authenticity perceptions.

Response:Thank you for this insightful comment. We agree that ritualized experiences may plausibly contribute to tourists’ authenticity perceptions, and that this potential relationship deserves clearer theoretical consideration.

In the revised manuscript, we have further clarified the rationale for treating ritualized experiences and perceived authenticity as two conceptually distinct but complementary antecedent domains. Ritualized experiences primarily capture the performative and structural characteristics of the tourism experience, including distinctiveness, commitment, ceremonial order, and non-functionality, whereas perceived authenticity reflects tourists’ cognitive and experiential evaluations of whether the heritage experience is historically credible, socially meaningful, and personally genuine. Accordingly, the present study focuses on their differentiated and configurational associations with tourists’ willingness to support ICH inheritance rather than assuming a specific causal ordering between the two constructs.

At the same time, we acknowledge that ritualized experiences may shape authenticity perceptions in some contexts. We have therefore added a discussion of this plausible sequential relationship in the theoretical development section and clarified that the present cross-sectional design does not allow us to establish whether ritualized experiences precede perceived authenticity. We have also identified this relationship as an important direction for future research, for example through longitudinal or mediation-based designs.

We believe that these revisions strengthen the theoretical integration of the two constructs while preserving the current study’s focus on their complementary net and configurational roles.

3.H3 should also be reformulated in set-theoretic terms because fsQCA identifies sufficient configurations rather than conventional “positive effects.”

Response:Thank you for this important comment. We agree that the original wording of H3 relied too heavily on conventional linear-effect terminology and did not adequately reflect the set-theoretic logic of fsQCA. In the revised manuscript, we have therefore reformulated H3 to emphasize sufficient configurations associated with both high and low levels of tourists’ willingness to support ICH inheritance.

Specifically, H3 has been revised as follows:Different configurations of ritualized experiences and perceived authenticity constitute sufficient pathways associated with high and low tourists’ willingness to support ICH inheritance, and the configurations associated with low willingness need not be the inverse of those associated with high willingness.

This revision better reflects the logic of fsQCA, which focuses on sufficient combinations of conditions rather than conventional independent positive or negative effects. It also aligns with the configurational principle of asymmetry, whereby the conditions associated with the presence of an outcome are not necessarily the mirror image of those associated with its absence.

4.The fsQCA procedure needs stronger justification and robustness assessment, particularly regarding the use of the 95th, 50th, and 5th percentile calibration anchors and the case-frequency threshold of 1 for a sample of 390. Please provide justification for these choices and conduct sensitivity analyses using alternative calibration anchors and higher frequency/consistency thresholds to demonstrate the robustness of the identified configurations.

Response:Thank you for this important comment. We agree that the calibration anchors and truth-table thresholds require clearer justification and that sensitivity analyses are necessary to assess the robustness of the configurational results.

In the revised manuscript, we have first clarified the rationale for the original calibration procedure. Because no externally established substantive calibration benchmarks were available for the constructs measured in this study, we adopted percentile-based direct calibration, using the 95th, 50th, and 5th percentiles as the thresholds for full membership, the crossover point, and full non-membership, respectively.

We further conducted sensitivity analyses using alternative calibration anchors and more stringent truth-table thresholds. Specifically, the calibration anchors were changed from the 95th, 50th, and 5th percentiles to the 90th, 50th, and 10th percentiles. In addition, the case-frequency threshold was increased from 1 to 3 and, in a more stringent specification, to 5, while the raw-consistency threshold was increased from 0.80 to 0.85, with the PRI-consistency threshold retained at 0.75.

The sensitivity analyses showed that the substantive configurational pattern remained stable under these alternative specifications. Increasing the case-frequency threshold mainly excluded low-frequency truth-table rows, while the configurations supported by larger numbers of cases remained. In particular, the low-willingness configuration characterized by the joint absence of the major experiential conditions remained stable under both the frequency-3 and frequency-5 specifications. The principal configurational structure associated with high willingness also remained substantively consistent, although some low-frequency combinations were excluded under the more stringent thresholds.

These additional analyses indicate that the main configurational conclusions are not driven solely by the original calibration anchors or truth-table thresholds. We have added the methodological justification and robustness results to the revised manuscript.

5.The contrast between the SEM and fsQCA results should be discussed more explicitly. In particular, commitment and constructive authenticity are not significant in the SEM model, yet they appear as relevant conditions in some configurational solutions. Please explain how these conditions can be configurationally important despite lacking significant independent net effects.

Response:Thank you for this important comment. We agree that the relationship between the SEM and fsQCA findings requires clearer explanation. In the revised manuscript, we have explicitly clarified that the two approaches address different analytical questions.

SEM estimates the independent net association of each predictor with tourists’ willingness to support ICH inheritance while accounting for the other predictors, whereas fsQCA examines whether conditions become relevant as part of sufficient configurations. Therefore, the nonsignificant SEM coefficients for commitment and constructive authenticity should not be interpreted as evidence that these conditions are unimportant. Rather, their presence in particular fsQCA solutions indicates configurational relevance: they may contribute to the outcome when combined with complementary ritualized-experience or authenticity conditions, even though they do not exhibit statistically significant independent net associations.

We have added this explanation to the Discussion section and further clarified in the Theoretical Implications section that the difference between the SEM and fsQCA results reflects the complementarity of net-effect and configurational perspectives rather than a contradiction between the two methods.

6.The causal language should be moderated throughout the manuscript. The cross-sectional and self-reported nature of the data does not establish temporal or causal relationships, and the term “causal asymmetry” in fsQCA should not be interpreted as experimental causality.

Response:Thank you for this important comment. We agree that the causal language in the manuscript should be moderated because the study is based on cross-sectional and self-reported data, which do not permit strong temporal or causal inference. In the revised manuscript, we have systematically replaced causal expressions such as “influence,” “effect,” “enhance,” “generate,” “foster,” “determine,” and “lead to” with more cautious terms such as “association,” “net association,” “configurational relevance,” and “configurations associated with the outcome,” where appropriate. We have also revised the SEM results, fsQCA interpretation, Discussion, Practical Implications, and Conclusion to ensure that the findings are presented as associations rather than causal effects.

We have further clarified the meaning of “causal asymmetry” in the fsQCA analysis. In the revised manuscript, this term is explicitly used in the set-theoretic sense, indicating that configurations associated with the presence of an outcome need not be the inverse of those associated with its absence; it does not imply experimental or temporal causality.

7.Please provide more information on the sampling strategy, the distribution of questionnaires across the study sites, the basis for determining the sample size, and the exact data-collection period. The timing should also be clearly consistent with the reported ethics approval date.

Response:Thank you for this important comment. We agree that the sampling and data-collection procedures required further clarification. In the revised manuscript, we have expanded Section 3.3 to provide additional information on the sampling strategy, sample distribution across the study sites, sample-size adequacy, and the exact data-collection period.

Specifically, we now clarify that data were collected from November 18, 2025 to January 8, 2026 using an on-site non-probability sampling strategy. A total of 425 questionnaires were collected, including 125 in Yongdeng County, 198 in Tongwei County, and 102 in Tianshui, of which 390 were retained as valid. We further added a sample-size adequacy assessment, noting that the final sample exceeded the commonly used ratio of 10 observations per measured item for SEM. The reported data-collection period also occurred after the ethics approval date of November 8, 2025. These revisions improve the transparency of the sampling and data-collection procedures.

8.The common method bias tests do not completely rule out the possibility of shared-method effects. Please interpret the results more cautiously and acknowledge the limitations of relying on single-source self-reported data.

Response:Thank you for this important comment. We agree that the statistical tests used to assess common method bias cannot completely rule out the possibility of shared-method effects. In the revised manuscript, we have therefore moderated our interpretation of the common method bias assessment.

Specifically, we revised the original statement to clarify that the results suggest that common method bias is unlikely to constitute a major threat to the findings, while the possibility of residual shared-method variance cannot be completely excluded. This more cautious wording avoids overstating the ability of Harman’s single-factor test and the single-factor measurement model to eliminate potential common method bias.We have also explicitly acknowledged the limitation of relying on single-source self-reported data in Section 5.4, Limitations and Future Research.

9.The outcome construct requires further justification because the four items capture somewhat heterogeneous forms of support, including recommendation, continued attention, participation, and the contribution of time or money. Please explain more clearly why these items can be treated as indicators of a single construct.

Response:Thank you for this important comment. We agree that the four outcome items capture different forms of heritage-supportive intention and therefore require clearer conceptual justification. In the revised manuscript, we have clarified that these items are not intended to represent identical behaviours, but rather complementary manifestations of a broader willingness to support ICH inheritance.

Specifically, recommendation and continued attention reflect communicative and attentional support, participation reflects engagement intention, and willingness to contribute time or financial resources reflects resource-based support. Despite these differences in form, all four items share a common underlying orientation toward supporting the continuity of ICH rather than actual heritage inheritance or intergenerational transmission. We have added this clarification to Section 3.2, Measurement Scales, to strengthen the conceptual basis for treating the four items as indicators of a single construct.

10.The practical implications should be framed as evidence-informed suggestions rather than as proven intervention effects. Please moderate prescriptive language and avoid implying that the proposed managerial actions will necessarily produce the desired outcomes.

Response:Thank you for this helpful comment. We agree that the practical implications should be presented as evidence-informed suggestions rather than as proven intervention effects. In the revised manuscript, we have moderated the prescriptive language throughout the Practical Implications section.

Specifically, expressions such as “should strengthen,” “should improve,” and “should adopt” have been revised to more cautious formulations such as “may consider,” “may benefit from,” and “greater attention may be given to.” We have also revised the concluding statements to avoid implying that the proposed managerial actions will necessarily produce specific outcomes. The revised section now presents these recommendations as practical implications informed by the observed associations and configurational patterns rather than as established causal interventions.

11.The low-willingness configuration should be interpreted cautiously until sensitivity analyses demonstrate its robustness.

Response:Thank you for this important comment. We agree that the low-willingness configuration should be interpreted cautiously. In the revised manuscript, we have moderated the wording used to describe the low-outcome solution and avoided presenting it as a definitive or causal explanation.

Specifically, we now state that the identified low-willingness configuration is associated with the concurrent absence of multiple key experiential conditions and should be interpreted cautiously because only one low-outcome configuration was identified. In addition, the newly added robustness assessment shows that this configuration remained stable under alternative analytical specifications, providing additional support for its robustness.

12. The limitations section should additionally acknowledge non-probability sampling, single-source self-report data, and possible heterogeneity across Shehuo locations.

Response: Thank you for this important comment. We agree that these methodological limitations should be made more explicit. In the revised manuscript, we have expanded the Limitations and Future Research section to acknowledge that the on-site non-probability sampling strategy may limit sample representativeness, that the use of single-source self-reported data may introduce response bias and residual shared-method effects, and that potential heterogeneity across the three Shehuo study locations was not explicitly examined. We have also suggested that future research employ more systematic sampling strategies, incorporate multiple data sources, and further examine cross-location heterogeneity.

13.A final round of language editing and formatting consistency checks is recommended throughout the manuscript.

Response:Thank you for this helpful suggestion. We have conducted a comprehensive language and formatting review of the revised manuscript. Specifically, we standardized the terminology used for the outcome construct as “tourists’willingness to support ICH inheritance,”corrected inconsistent abbreviations and residual wording, and standardized the formatting of section headings, figure and table captions, punctuation, spacing, and capitalization.

We also corrected grammatical and typographical errors and ensured greater consistency in English spelling and academic expression throughout the manuscript. These revisions were made across the Abstract, main text, tables, figure captions, and notes to improve the overall clarity and presentation of the manuscript.

Reviewer #3:

1.Ethics approval number. The manuscript names the approving body (College of Tourism, Northwest Normal University IRB) but does not provide a specific approval number. PLOS ONE's reporting guidelines ask authors to "include the approval number if one was obtained." Please either insert the approval number or explicitly state that no numbered approval was issued for this study.

Response:Thank you for this helpful suggestion. We have clarified the ethics statement in the revised manuscript. The study was reviewed and approved by the Institutional Review Board of the College of Tourism, Northwest Normal University on November 8, 2025. As no numbered approval was issued for this study, we have now explicitly stated this in the Ethics Statement.

2.fsQCA calibration anchors. The 95 %/5 % percentile anchors are distribution-driven rather than theory- or logic-driven, which remains debated in the QCA literature. The authors provide a justification, but it would strengthen the manuscript to note this choice as a limitation in the Discussion, or to flag that future replications should test robustness against theory-based anchor points.

Response:Thank you for this helpful suggestion. We have added a brief limitation regarding the percentile-based calibration strategy in Section 5.4. We now clarify that the calibration anchors used in the fsQCA are distribution-driven rather than theory-based. Although the robustness analyses using alternative percentile anchors yielded substantively consistent results, we also note that future studies should further examine the stability of the findings using theoretically grounded calibration criteria where available.

3.Distinction between net-effect and configurational logic. The retention of H3 is well defended. To pre-empt reader confusion, I suggest adding one explicit sentence in the Discussion clarifying that a non-significant net effect in the SEM does not imply the condition is unimportant in a configuration—that is, the two analytical lenses answer different questions. This would make the relationship between H1/H2 and H3 fully transparent.

Response:Thank you for this helpful suggestion. We have added an explicit clarification in the Discussion section to distinguish the analytical logic of SEM and fsQCA. Specifically, we now explain that SEM assesses the independent net association of each predictor with the outcome, whereas fsQCA examines the configurational relevance of conditions within sufficient combinations. We further clarify that a nonsignificant SEM coefficient does not imply that a condition is unimportant in a configurational solution.

Attachment Submitted filename: Respond to Reviewers1.docx

# Editor report: Decision Letter 2

**DOI:** 10.1371/journal.pone.0358987.r005

**Authors:** Bo Pu

8 Sep 2026

Ritualized Experiences and Perceived Authenticity in Shaping Tourists’ Willingness to Support Intangible Cultural Heritage Inheritance

PONE-D-26-28004R2

Dear Dr. Song,

We’re pleased to inform you that your manuscript has been judged scientifically suitable for publication and will be formally accepted for publication once it meets all outstanding technical requirements.

Within one week, you’ll receive an e-mail detailing the required amendments. When these have been addressed, you’ll receive a formal acceptance letter and your manuscript will be scheduled for publication.

An invoice will be generated when your article is formally accepted. Please note, if your institution has a publishing partnership with PLOS and your article meets the relevant criteria, all or part of your publication costs will be covered. Please make sure your user information is up-to-date by logging into Editorial Manager at Editorial Manager® and clicking the ‘Update My Information' link at the top of the page. For questions related to billing, please contact billing support.

If your institution or institutions have a press office, please notify them about your upcoming paper to help maximize its impact. If they’ll be preparing press materials, please inform our press team as soon as possible -- no later than 48 hours after receiving the formal acceptance. Your manuscript will remain under strict press embargo until 2 pm Eastern Time on the date of publication. For more information, please contact onepress@plos.org.

Kind regards,

Bo Pu, Ph.D.

Academic Editor

PLOS One

Additional Editor Comments (optional):

thanks for your hard work toward this manuscript.

Reviewers' comments:

# Editor report: Acceptance letter

**DOI:** 10.1371/journal.pone.0358987.r006

**Authors:** Bo Pu

PONE-D-26-28004R2

PLOS One

Dear Dr. Song,

I'm pleased to inform you that your manuscript has been deemed suitable for publication in PLOS One. Congratulations! Your manuscript is now being handed over to our production team.

At this stage, our production department will prepare your paper for publication. This includes ensuring the following:

* All references, tables, and figures are properly cited

* All relevant supporting information is included in the manuscript submission,

* There are no issues that prevent the paper from being properly typeset

You will receive further instructions from the production team, including instructions on how to review your proof when it is ready. Please keep in mind that we are working through a large volume of accepted articles, so please give us a few days to review your paper and let you know the next and final steps.

Lastly, if your institution or institutions have a press office, please let them know about your upcoming paper now to help maximize its impact. If they'll be preparing press materials, please inform our press team within the next 48 hours. Your manuscript will remain under strict press embargo until 2 pm Eastern Time on the date of publication. For more information, please contact onepress@plos.org.

You will receive an invoice from PLOS for your publication fee after your manuscript has reached the completed accept phase. If you receive an email requesting payment before acceptance or for any other service, this may be a phishing scheme. Learn how to identify phishing emails and protect your accounts at https://explore.plos.org/phishing.

If we can help with anything else, please email us at customercare@plos.org.

Thank you for submitting your work to PLOS One and supporting open access.

Kind regards,

PLOS One Editorial Office Staff

on behalf of

Dr. Bo Pu

Academic Editor

PLOS One
